# Diaphragmatic Ultrasound in Fibrosing Interstitial Lung Disease: A Systematic Review of Current Evidence

**DOI:** 10.3390/diagnostics16172759

**Published:** 2026-08-28

**Authors:** Sanjeewa Patabendige, Casper Falster, Henrik Z. Langkilde, Stefan M. W. Harders, Elisabeth Bendstrup, Søren Helbo Skaarup, Michael T. Durheim, Jesper Rømhild Davidsen

**Affiliations:** 1 Odense Respiratory Research Unit (ODIN), Department of Clinical Research, University of Southern Denmark, Odense University Hospital, 5000 Odense, Denmark; 2 South Danish Center for Interstitial Lung Diseases (SCILS), Department of Respiratory Medicine, Odense University Hospital, 5000 Odense, Denmark; 3 Pulmo-Rheuma Frontline Center (PURE), Department of Respiratory Medicine, Odense University Hospital, 5000 Odense, Denmark; 4 Research Unit of Rheumatology, Department of Clinical Research, University of Southern Denmark, Odense University Hospital, 5000 Odense, Denmark; 5 Department of Radiology, University of Southern Denmark, Odense University Hospital, 5000 Odense, Denmark; 6 Center for Rare Lung Diseases, Department of Respiratory Diseases and Allergy, Aarhus University Hospital, 8200 Aarhus, Denmark; 7 Department of Clinical Medicine, Aarhus University, 8200 Aarhus, Denmark; 8 Duke Clinical Research Institute, Durham, NC 27710, USA; 9 Department of Respiratory Medicine, Oslo University Hospital—Rikshospitalet, 0372 Oslo, Norway

**Keywords:** fibrosing interstitial lung disease, diaphragmatic ultrasound, idiopathic pulmonary fibrosis, progressive pulmonary fibrosis, high resolution computed tomography

## Abstract

**Background/Objectives**: Fibrosing interstitial lung disease (F-ILD) is associated with progressive respiratory impairment and substantial symptom burden. Diaphragmatic ultrasound (DUS) is a non-invasive method for assessing diaphragmatic structure and function, but its clinical role in F-ILD remains uncertain. This systematic review evaluated the available evidence on DUS in adults with F-ILD. **Methods**: This systematic review was conducted and reported according to the PRISMA guidelines. MEDLINE, Embase, CINAHL, and the Cochrane Library were searched and observational studies evaluating DUS in adults with F-ILD were included. Risk of bias was assessed using the QUADAS-2, and outcome-level certainty of evidence was evaluated by GRADE framework. **Results**: Six cross-sectional observational studies involving 232 participants were included. Diaphragmatic excursion (DE) was assessed in all six studies, while diaphragm thickness (DT) and thickening fraction (TF) were evaluated in four. Some studies reported abnormalities in DUS parameters during deep breathing and cross-sectional associations with pulmonary function, exercise capacity, dyspnoea, or radiological severity. Substantial heterogeneity in study populations, ultrasound protocols, and outcome reporting precluded meta-analysis. The certainty of evidence was very low. **Conclusions**: Current evidence suggests that DUS may detect diaphragmatic abnormalities in patients with F-ILD and may have future complementary value alongside established clinical assessments. However, the evidence is limited, heterogeneous, and of very low certainty and does not establish diagnostic accuracy, prognostic value, or usefulness for longitudinal monitoring. Accordingly, DUS cannot currently be recommended for routine clinical implementation in F-ILD, and prospective longitudinal validation is required.

## 1. Introduction

Fibrosing interstitial lung disease (F-ILD) comprise a heterogeneous group of disorders with diverse etiologies and clinical phenotypes that are unified by the progressive fibrosing remodeling of the lung parenchyma, ultimately resulting in impaired gas exchange, declining pulmonary function, and increasing respiratory disability [1,2,3,4,5]. This group includes idiopathic pulmonary fibrosis (IPF), the prototypical progressive fibrosing ILD, as well as non-IPF F-ILD that may develop a progressive pulmonary fibrosis (PPF) phenotype despite differences in their underlying pathogenic mechanisms [1,2,5]. Accurate assessment of disease severity and progression is fundamental to patient management and relies on a combination of clinical evaluation, pulmonary function testing (PFT), and serial high-resolution computed tomography (HRCT) [2,5]. However, these methods are resource-intensive, require patient cooperation, expose patients to cumulative radiation with associated oncogenic risks, and may thus limit the feasibility of frequent longitudinal evaluation [6]. Consequently, there is growing interest in complementary, non-invasive methods capable of providing additional and functional information during disease evaluation and follow-up.

Diaphragmatic ultrasound (DUS) has recently gained attention as a promising bedside and non-invasive method for assessing diaphragmatic structure and function in patients with F-ILD [7,8,9]. The method enables real-time evaluation of diaphragmatic functional abnormalities through measurements such as diaphragmatic excursion and thickening fraction [7,8,9]. In patients with F-ILD, progressive fibrosing remodeling alters respiratory mechanics, increases inspiratory load, and may contribute to diaphragmatic dysfunction through reduced mobility and impaired contractile performance [7,9]. These pathophysiological changes have prompted increasing interest in the potential role of DUS as a functional imaging modality in this patient population.

Preliminary observational studies have reported reduced diaphragmatic excursion and altered diaphragmatic contractility in patients with F-ILD compared with healthy individuals, with some studies suggesting associations between diaphragmatic dysfunction, impaired pulmonary function, reduced exercise capacity, and greater symptom burden [7,8,9]. However, the available evidence remains limited, and the potential clinical role of DUS in patients with F-ILD has not yet been clearly established.

To our knowledge, the current evidence regarding the use of DUS in patients with F-ILD has not previously been systematically synthesized. A comprehensive evaluation of the available literature is therefore warranted to summarize the current evidence, critically appraise the methodological quality of published studies, identify existing knowledge gaps, and highlight priorities for future research.

Our previous systematic review evaluated the broader applicability of thoracic ultrasound (TUS) in F-ILD, focusing primarily on pulmonary and pleural findings such as B-lines and pleural-line irregularities [1]. It did not specifically synthesise evidence concerning diaphragmatic structure or function. The present review addresses a distinct research question by focusing exclusively on DUS-specific parameters, their associations with clinically relevant outcomes, and the methodological quality and certainty of the available evidence. Importantly, there was no overlap between the primary studies included in the previous review and those included in the present review.

Accordingly, we conducted this systematic review to critically evaluate the current evidence regarding the use of DUS in patients with F-ILD.

## 2. Materials and Methods

### 2.1. Registration of Study Protocol

The protocol for this systematic review was registered in the International Prospective Register of Systematic Reviews (PROSPERO) prior to study initiation to ensure methodological transparency and avoid duplication of ongoing reviews. Any amendments made during the review process were documented in the PROSPERO record (Supplementary S1).

### 2.2. Search Strategy

This systematic review was conducted following the recommendations of the Preferred Reporting Items for Systematic Reviews and Meta-Analyses (PRISMA) guidelines (Supplementary S2) [10]. The review question was developed according to the Population, Intervention, Comparator, Outcome (PICO) framework:

**P**opulation—patients with F-ILD fulfilling classification criteria that were relevant at the time of the study.

**I**ntervention—various DUS methods employed to evaluate diaphragmatic dysfunction in the included studies.

**C**omparator—reference clinical, physiological, or imaging assessments used in the individual studies.

**O**utcome—DUS parameters (e.g., diaphragmatic excursion, diaphragm thickness, and thickening fraction) and their reported associations with HRCT, PFT pulmonary, exercise capacity, symptom burden, or other clinically relevant outcomes.

A comprehensive literature search was performed in MEDLINE, Embase, CINAHL, and the Cochrane Library from database inception until the final search date. The search strategy was developed in collaboration with an experienced research librarian and subsequently reviewed and approved by all authors (Tables S1 and S2).

Both subject headings and subheadings for ILD, ultrasound, and diagnosis were employed. The initial search was performed on 5 June 2023, with updates on 27 September 2023, and 15 February 2025, to capture all relevant publications in the field.

Controlled vocabulary (including MeSH and Emtree terms) together with free-text keywords relating to fibrosing interstitial lung disease, diaphragm, diaphragmatic ultrasound, and ultrasonography were combined using Boolean operators. To maximize retrieval, backward and forward citation tracking of all eligible studies was additionally performed. No restrictions on publication date were applied; however, only studies published in English were considered eligible.

### 2.3. Study Selection

All retrieved records were imported into the web-based platform Covidence for study management, where duplicate records were identified and removed using both automated and manual procedures.

Study selection was conducted in two stages. First, two reviewers independently screened titles and abstracts based on predefined eligibility criteria. Second, full-text articles of potentially relevant studies were independently assessed by the same reviewers, who were blinded to each other’s decisions. When clarification was required, corresponding authors were contacted to obtain additional information relevant to eligibility assessment. Discrepancies between reviewers were resolved by discussion, with arbitration by the main supervisor when consensus could not be achieved.

The search was restricted to full-text articles published in English. Grey literature, including theses, preprints, trial registries, and unpublished studies, was not systematically searched. Conference abstracts were excluded because they provided insufficient information for eligibility assessment, data extraction, and risk-of-bias evaluation. Protocols, reviews, case reports, case series, book chapters, and publications without original clinical data were also excluded.

A detailed list of studies excluded after full-text review is provided in Table S3. For studies reported in multiple publications, the version presenting the most complete and relevant data was included in the final analysis.

### 2.4. Eligibility Criteria

Studies were eligible if they reported original clinical data on the use of DUS to assess diaphragmatic structure or function in adults with F-ILD. For this review, F-ILD was operationally defined as an ILD with an explicitly identified fibrotic component, as reported by the study authors and diagnosed according to the clinical, radiological, and/or multidisciplinary diagnostic framework applicable when the study was conducted. Eligible diagnoses included IPF and clearly specified non-IPF fibrotic ILDs, such as CTD-ILD with pulmonary fibrosis, fNSIP, fHP, fibrotic sarcoidosis, unclassified fibrotic IIP, and CPFE.

IPF was regarded as an established fibrotic ILD and was not required to fulfil separate PPF criteria. Studies of non-IPF F-ILD were not retrospectively required to meet the current PPF definition, particularly when they predated this framework. Eligibility was independently assessed by two reviewers based on the diagnosis and diagnostic criteria reported in each primary study.

Studies were excluded when the ILD subtype or the presence of pulmonary fibrosis could not be sufficiently established from the reported study population. Study protocols, reviews, case reports, case series, conference abstracts, book chapters, and other publications that did not report original clinical data were also excluded.

### 2.5. Data Extraction

Data extraction was independently performed by two reviewers using a standardized data extraction form developed prior to study initiation. Extracted variables included study characteristics, patient demographics, F-ILD subtype, ultrasound methodology, diaphragmatic ultrasound parameters, comparator assessments, and reported clinical outcomes.

Whenever relevant information was incomplete or unclear, the corresponding authors were contacted for clarification.

### 2.6. Quality Assessment

Methodological quality and risk of bias were independently evaluated by two reviewers using QUADAS-2, as prespecified in the prospectively registered PROSPERO protocol [11]. Although QUADAS-2 was developed for diagnostic accuracy studies, it was applied pragmatically because DUS was evaluated as an index assessment in relation to clinical, physiological, functional, or imaging comparators. The “reference standard” domain was interpreted as assessing the validity and appropriateness of the comparator used for the specific association under investigation, including pulmonary function testing, HRCT-based fibrosis assessment, exercise capacity, or symptom measures. These comparators were not considered uniform gold standards for diaphragmatic dysfunction, and the QUADAS-2 assessment was not used to infer the diagnostic accuracy of DUS.

Any disagreements between reviewers were resolved through discussion, with arbitration by a main supervisor when necessary.

### 2.7. Evaluation of Certainty of Evidence

Certainty of evidence was assessed separately for diaphragm displacement/excursion, diaphragm thickness, and thickening fraction using the GRADE framework (Grading of Recommendations Assessment, Development and Evaluation) and the approach described by Murad et al. [12]. As quantitative pooling was not possible, judgements were based on the overall body of evidence contributing to each outcome rather than on pooled estimates. Risk of bias, indirectness, imprecision, inconsistency, publication bias, and potential upgrading criteria were considered.

### 2.8. Data Synthesis

A meta-analysis was planned but was not considered appropriate because of substantial clinical and methodological heterogeneity. Studies differed in patient populations and F-ILD subtypes, ultrasound techniques, assessed hemidiaphragm, patient positioning, breathing manoeuvres, acquisition protocols, and DUS parameters, including excursion or displacement, thickness, and thickening fraction. Comparator outcomes varied across pulmonary function, exercise capacity, dyspnoea, anthropometric measures, and radiological severity. Findings were also reported using different formats, including group comparisons, correlations, and cut-off values, without sufficiently comparable effect estimates. Therefore, quantitative pooling was considered inappropriate, and the findings were synthesised narratively according to DUS parameters and associated clinical outcomes.

## 3. Results

**Figure 1:**
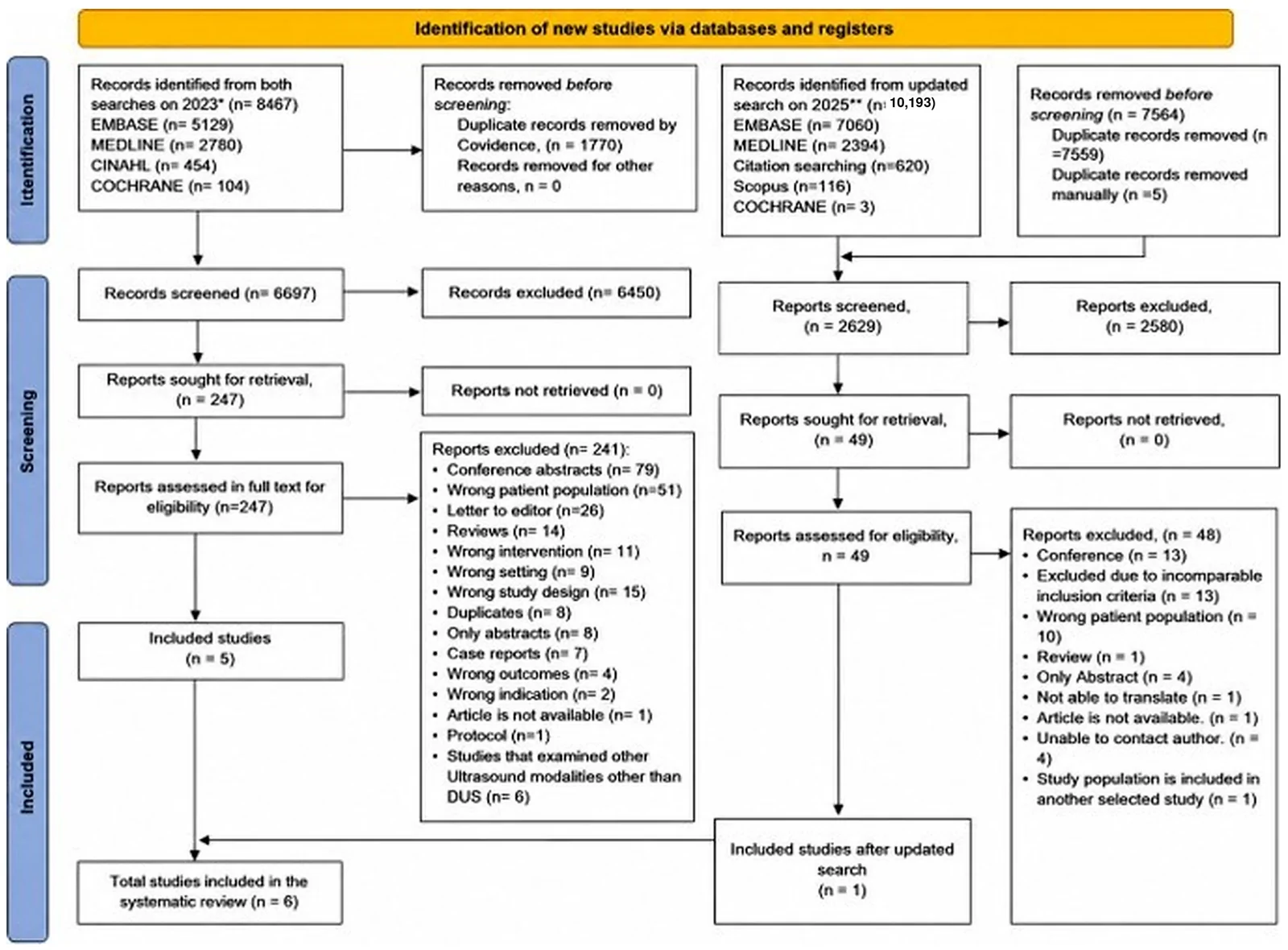
PRISMA 2020 flow diagram showing the identification, screening, eligibility assessment, and inclusion of studies in the systematic review. PRISMA, Preferred Reporting Items for Systematic Reviews and Meta-Analyses. * A comprehensive literature search was initially conducted on 5 June 2023, and an updated search was performed on 27 September 2023. ** Identification of new studies was carried out on 15 February 2025, including full database searches and citation tracking (both forward and backward citation searching).

Database searches identified 18,660 records: 8467 from the original search conducted in 2023 and 10,193 from the updated search conducted in 2025. In the original search, 6697 records remained after duplicate removal, of which 247 underwent full-text assessment and five studies were included. In the updated search, 2629 records remained after duplicate removal, of which 49 underwent full-text assessment. One additional eligible study was identified, resulting in six studies in the final review [13,14,15,16,17,18]. The study selection process is presented in the PRISMA flow diagram (Figure 1).

### 3.1. Summary of Included Studies (Table 1)

All six studies enrolled patients with IPF [13,14,15,16,17,18], while three also included participants with other forms of F-ILD [13,15,18]. Across the included studies, sample sizes ranged from a minimum of 12 to a maximum of 82 patients, totaling 232 patients across the various F-ILD categories.

**Table 1:** Characteristics of the six included studies.

Study	Country	Design/Setting	Inclusion Period	Population	*n*	Age (Years)	Key Exclusion Criteria
Bernardinello et al. (2023) [13]	Italy	Cross-sectional case–control; single centre	Mar.–Oct. 2020	CTD-ILD and IPF	82	Median 70	Emphysema or COPD; active infection; previous or recent abdominal/thoracic surgery; prednisolone-equivalent dose ≥ 25 mg/day; neuromuscular disease
Boccatonda et al. (2019) [14]	Italy	Cross-sectional case–control; single centre	Not specified	IPF	12	Mean 66.5	Ongoing pulmonary infection; neuromuscular disorders associated with pulmonary fibrosis
He et al. (2014) [15]	China	Cross-sectional case–control; single centre	2009–2012	CPFE, COPD and IPF	43	CPFE: mean 65.57; IPF: mean 63.54	BMI < 18.5 or >30 kg/m^2^
Kısmet et al. (2023) [16]	Türkiye	Cross-sectional case–control; single centre	Jan. 2016–Feb. 2021	IPF	41	Mean 72.1	COPD or CPFE; systemic corticosteroid use; previous pleural disease; upper abdominal or thoracic surgery
Milesi et al. (2023) [17]	France	Cross-sectional case–control; single centre	Dec. 2020–Feb. 2023	IPF	24	Mean 66	COPD; cystic fibrosis/bronchiectasis; autoimmune disease with evidence of muscle involvement; myopathy; active infection; upper abdominal or thoracic surgery (except diagnostic surgery for IPF); exacerbation or rehabilitation within 2 months
Santana et al. (2019) [18]	Brazil	Cross-sectional case–control; single centre	Feb. 2014–Feb. 2016	fHP, CTD-ILD, unclassified IIP, fNSIP, IPF and fibrotic sarcoidosis	30	Mean 49	COPD; active infection; neuromuscular disease; CTD-ILD with clinical or laboratory evidence of myositis/myopathy

BMI, body mass index; COPD, chronic obstructive pulmonary disease; CPFE, combined pulmonary fibrosis and emphysema; CTD-ILD, connective tissue disease-associated interstitial lung disease; fHP, fibrotic hypersensitivity pneumonitis; fNSIP, fibrotic nonspecific interstitial pneumonia; IIP, idiopathic interstitial pneumonia; IPF, idiopathic pulmonary fibrosis.

All studies were either explicitly described as cross-sectional or judged to be cross-sectional based on this review criteria. The ILD subtypes included varied considerably across the studies. Apart from IPF, the included F-ILD subtypes were connective tissue disease related ILD (CTD-ILD) [13,18], combined pulmonary fibrosis and emphysema (CPFE) [15], unclassified idiopathic interstitial pneumonias (IIP) [18], fibrotic nonspecific interstitial pneumonia (fNSIP) [18], fibrotic sarcoidosis [18] and fibrotic hypersensitivity pneumonitis (fHP) [18].

### 3.2. DUS Findings Retrieved from the Included Studies (Figure 2/Table 2)

Several DUS parameters were used to assess the diaphragm across the six studies, with most studies employing more than one parameter. For consistency, movement-based measurements originally described as diaphragmatic displacement, mobility, motion, or excursion were collectively reported as diaphragmatic excursion (DE). DE was assessed in all six studies [13,14,15,16,17,18], while diaphragm thickness (DT) and thickening fraction (TF) were evaluated in four studies [13,16,17,18]. Only Bernardinello et al. reported an explicit TF cut-off of <30% to define diaphragmatic dysfunction [13] Only four studies assessed the right hemi diaphragm [13,14,16,18], whereas 2 studies evaluated both hemi diaphragms [15,17].

**Figure 2:**
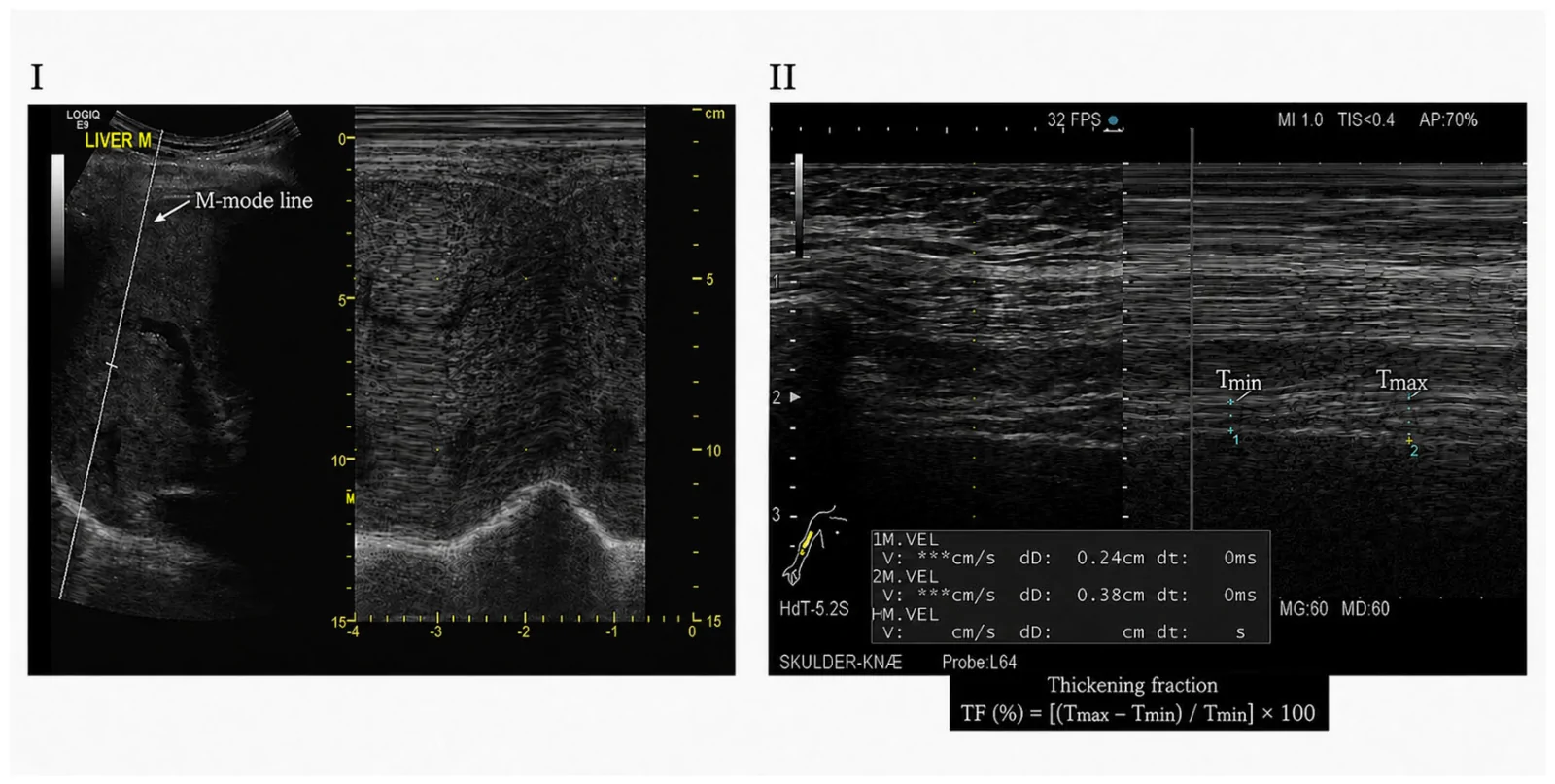
Different types of diaphragmatic ultrasound measurements. (**I**) B-mode image showing placement of the M-mode sampling line across the diaphragm, with the corresponding M-mode trace used to assess diaphragmatic excursion. (**II**) M-mode assessment of diaphragm thickness at minimum thickness and maximum thickness. Thickening fraction (TF) was calculated as: TF (%) = [(Tmax − Tmin)/Tmin] × 100.

**Table 2:** Other major findings in included studies.

Study	Ultrasound System and Transducer(s)	Probe Placement/Acoustic Window	Patient Position/Side	Mode and DUS Parameter(s)	Breathing Manoeuvre and Measurement Protocol	Cut-Off	Comparator(s)	Main Conclusion
**Bernardinello et al. (2023)** [13]	Portable SonoSite M-Turbo; convex C60xi, 2–5 MHz (DE); linear HFL38x, 6–13 MHz (DT)	Convex probe directed dorsocranially from the right anterior-to-midclavicular region; linear probe at the right mid-axillary line, perpendicular to the diaphragm, approximately at the 8th–10th intercostal spaces	Semi-recumbent (40° head-up); right hemidiaphragm	M-mode: DE (reported as displacement), minimum and maximum DT; TF calculated	Quiet breathing; mean of three consecutive measurements. Initial measurements assessed by two observers; remaining examinations performed by one operator	TF < 30% for diaphragmatic dysfunction	PFT and dyspnoea	Diaphragmatic dysfunction was present in 29% and was associated with moderate-to-severe dyspnoea. CTD-ILD showed lower DE than IPF and a higher prevalence of diaphragmatic dysfunction than controls; TF was associated with lung function only in CTD-ILD.
**Boccatonda et al. (2019)** [14]	Portable Aloka ProSound; 2–5 MHz convex probe	Right ascending subcostal scan between the anterior axillary and midclavicular lines	Supine; right hemidiaphragm	B-mode visualisation; M-mode measurement of DE (reported as mobility)	Three measurements at rest and after deep inspiration; same blinded operator; excursion measured from baseline to the apex of the frozen diaphragmatic curve	NR	PFT and anthropometric parameters	Diaphragmatic excursion was lower in patients with IPF than in healthy controls, particularly during deep inspiration.
**He et al. (2014)** [15]	Esaote MyLab30; 3.5 MHz convex transducer	Low anterior subcostal approach, coronal intercostal approach, or both; liver window on the right and splenic window on the left	Supine; both hemidiaphragms	Two-dimensional mode for localisation; M-mode measurement of DE (reported as diaphragmatic motion)	Quiet and deep breathing; three measurements per participant, with the best value retained; same experienced radiologist blinded to clinical information	NR	PFT and HRCT emphysema/fibrosis scores	Right DE during deep breathing correlated negatively with emphysema score; no correlation was observed with fibrosis score.
**Kısmet et al. (2023)** [16]	Esaote MyLab; 1–8 MHz convex transducer (DE); 3–13 MHz linear transducer (DT)	DE: anterior subcostal region between the midclavicular and anterior axillary lines. DT: zone of apposition between the anterior and mid-axillary lines near the costophrenic angle	Supine; right hemidiaphragm	Two-dimensional localisation; M-mode DE (reported as mobility); B-mode DT; TF calculated	Quiet breathing at FRC and maximal deep breathing at TLC; maximal inspiration sustained for ≥2 s; average of three effective measurements; measurements from two experienced radiologists recorded	NR	PFT, 6MWT, mMRC and total fibrosis score	Diaphragmatic function appeared preserved in mild-to-moderate restrictive IPF, with no association between DUS parameters, respiratory function or fibrosis extent.
**Milesi et al. (2023)** [17]	GE Vivid S60N; cardiac probe for DE (frequency NR); high-frequency 9L linear probe for DT	Right DE: subcostal between the midclavicular and anterior axillary lines, directed medially, cranially and dorsally. Left DE: subcostal or low intercostal between the anterior and mid-axillary lines. DT: zone of apposition between the anterior and mid-axillary lines	Seated; both hemidiaphragms	M-mode DE; B-mode DT; TF calculated	Quiet breathing, deep breathing and voluntary sniffing; measurements averaged over ≥3 breathing cycles; one experienced operator	NR	PFT, 6MWT and mMRC	DE and DT were reduced in patients with IPF compared with controls.
**Santana et al. (2019)** [18]	SonoSite NanoMaxx or M-Turbo; 2–5 MHz convex transducer (DE); 6–13 MHz linear transducer (DT)	DE: right anterior subcostal region between the midclavicular and anterior axillary lines, directed medially, cephalad and dorsally. DT: zone of apposition between the right anterior and mid-axillary lines	Semi-recumbent; right hemidiaphragm	Two-dimensional visualisation; M-mode DE (reported as mobility); two-dimensional DT; TF calculated	Quiet breathing at FRC and maximal deep breathing at TLC; maximal inspiration sustained for ≥2 s; ≥3 measurements averaged; one experienced physician	NR	PFT, dyspnoea, MRC and 6MWT	Patients with F-ILD demonstrated reduced DE and TF during deep breathing.

6MWT, 6-min walk test; CTD-ILD, connective tissue disease-associated interstitial lung disease; DE, diaphragmatic excursion; DT, diaphragm thickness; DUS, diaphragmatic ultrasound; F-ILD, fibrosing interstitial lung disease; FRC, functional residual capacity; HRCT, high-resolution computed tomography; IPF, idiopathic pulmonary fibrosis; mMRC, modified Medical Research Council; MRC, Medical Research Council; NR, not reported; PFT, pulmonary function testing; TF, thickening fraction; TLC, total lung capacity. For consistency, movement-based measurements reported by the primary studies as displacement, mobility, motion, or excursion are collectively presented as DE. Diaphragmatic dysfunction is written in full and refers to the study-specific categorical definition.

Furthermore, all studies used PFT as the reference standard to assess the correlation [13,14,15,16,17,18]. In addition to PFT, three studies also employed the 6-min walk test (6MWT) and dyspnoea scales, including the Medical Research Council (MRC) or modified Medical Research Council (mMRC) scale [16,17,18]. Other methods used to assess the correlation included anthropometric parameters [14], total fibrosis score from HRCT [16], and assessment of respiratory symptoms (dyspnea) quantified by the MRC scale [18]. The ultrasound methodology, comparator assessments, principal findings, and main conclusions of the included studies are summarised in Table 2.

### 3.3. QUADAS-2 Assessment (Figure 3)

The quality of the included studies was assessed using the QUADAS-2 tool [11], and most of the studies demonstrated concerns regarding the risk of bias. Five studies were judged to have a high risk of bias in the patient selection domain, while one study was rated as having an unclear risk. In the index test domain, four studies were considered to have a high risk of bias and two unclear risk. The reference standard domain was predominantly judged as having an unclear risk of bias across the included studies. Similarly, five studies were rated as having an unclear risk in the flow and timing domain. A detailed summary of the QUADAS-2 assessment is provided in Table S4, Figures S1 and S2.

**Figure 3:**
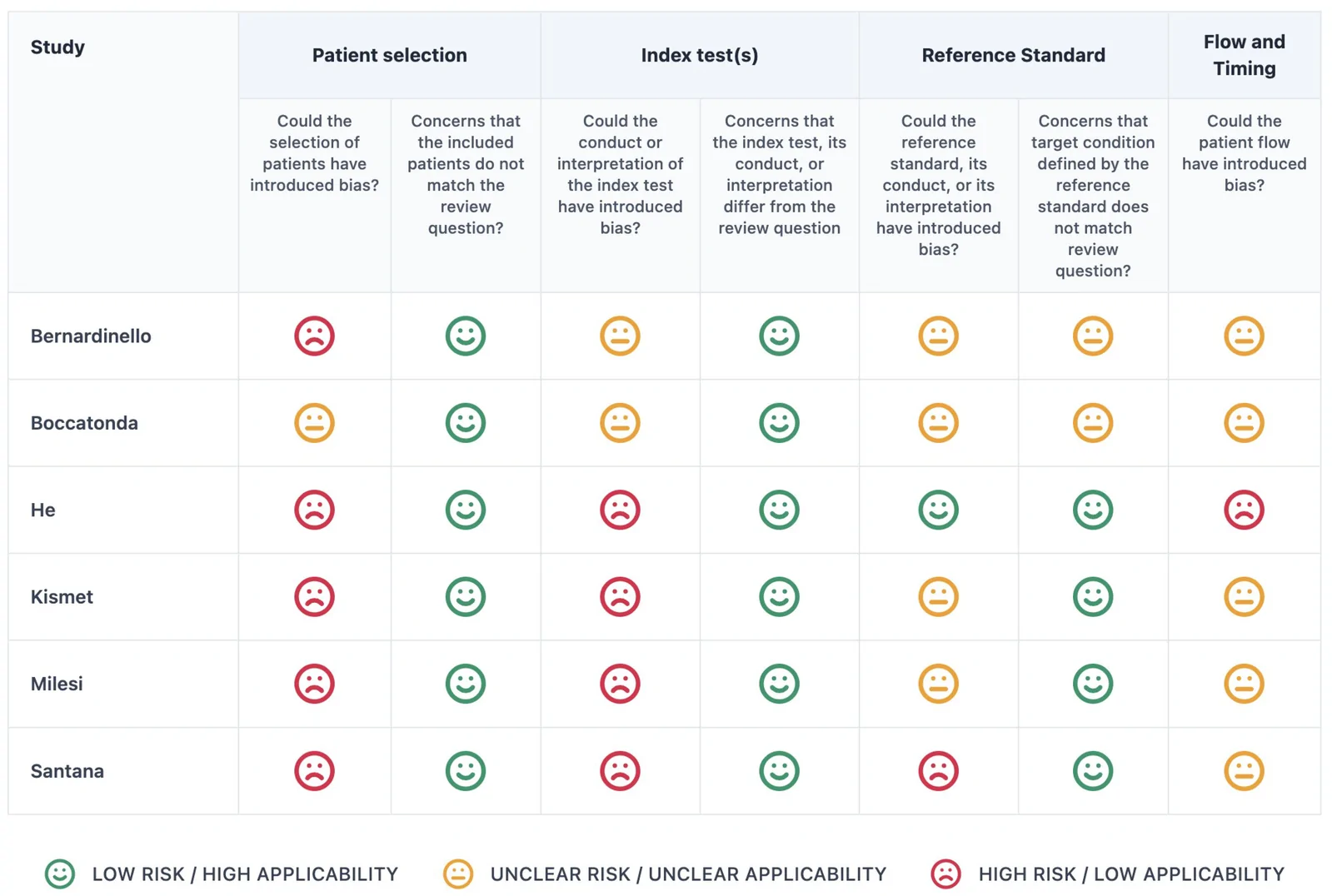
Summary of the risk-of-bias and applicability assessments for each included study using QUADAS-2.

### 3.4. GRADE Evaluation

Certainty was rated as very low for all three DUS outcomes. Observational evidence started at low certainty and was downgraded for serious methodological limitations and imprecision due to small sample sizes. Publication bias could not be formally assessed because fewer than 10 studies contributed to each outcome, and no upgrading criteria were identified. Outcome-specific judgements are presented in Table S5.

## 4. Discussion

### 4.1. Summary of Key Findings

To our knowledge, this is the first systematic review to comprehensively evaluating DUS in patients F-ILD. Six observational studies involving 232 patients met the inclusion criteria. Although methodological diversity precluded meta-analysis, several consistent findings emerged. Diaphragmatic excursion and diaphragm thickness were the most frequently assessed ultrasound parameters, and most studies reported impaired diaphragmatic function during deep inspiration. Furthermore, several studies demonstrated associations between DUS findings and pulmonary function, exercise capacity, dyspnea, and radiological disease severity. However, the certainty of evidence was very low, preventing firm conclusions regarding the routine clinical use of DUS.

### 4.2. Comparison with Existing Literature

The observed findings are physiologically plausible. Progressive fibrosing remodeling increases respiratory system stiffness and inspiratory workload, potentially impairing diaphragmatic mechanics and contractile function [19,20]. Chronic hypoxemia, systemic inflammation, physical deconditioning, and corticosteroid exposure may further contribute to respiratory muscle dysfunction [21,22,23,24]. Together, these mechanisms provide a biological rationale for assessing diaphragmatic function using ultrasound in patients with F-ILD.

Three previous studies investigated DUS in broader ILD populations but were excluded because they did not specifically evaluate patients with F-ILD or apply contemporary diagnostic criteria for progressive pulmonary fibrosis [7,8,9]. Although their findings generally support the present results, differences in study populations limit direct comparison.

### 4.3. Comparison Across the Included Studies

Despite differences in study design and ultrasound methodology, several observations were consistent across the included studies. Reduced diaphragmatic mobility during deep inspiration was the most frequently reported abnormality, while multiple studies demonstrated associations between DUS parameters and established markers of disease severity, including pulmonary function, exercise capacity, dyspnea, and radiological fibrosis [13,14,15,16,17,18].

The available evidence is limited primarily to cross-sectional associations. Although DUS parameters were associated with pulmonary function, exercise capacity, dyspnoea, and radiological severity in some studies, these associations do not establish diagnostic accuracy or demonstrate that DUS provides information beyond established clinical assessments. Furthermore, no included study evaluated whether DUS predicts clinical outcomes or reliably detects longitudinal disease progression or treatment response. Therefore, the diagnostic, prognostic, and monitoring value of DUS in F-ILD remains unestablished.

However, substantial diversity existed regarding patient populations, F-ILD subtypes, ultrasound acquisition protocols, breathing maneuvers, evaluated parameters, and reported outcomes [13,14,15,16,17,18]. Consequently, the principal limitation of the current literature is not the absence of positive findings, but the lack of methodological standardization. This diversity prevented quantitative synthesis and currently precludes conclusions regarding the diagnostic, prognostic, or monitoring value of DUS [13,14,15,16,17,18].

Considerable procedural variation was identified across the included studies, including differences in ultrasound systems, transducer types and frequencies, probe placement, patient positioning, assessed hemidiaphragm, ultrasound mode, breathing maneuvers, and the number and selection of repeated measurements. These factors may influence DUS measurements and limit direct comparisons across studies. Standardized acquisition and reporting protocols are therefore required before reproducible reference values and clinically meaningful thresholds can be established.

Clinical heterogeneity among the included studies also warrants consideration. The inclusion of patients with CPFE may have influenced the reported DUS findings because coexisting fibrosis and emphysema can produce different respiratory mechanics from F-ILD without emphysema [15]. Variations in F-ILD subtypes and the presence of emphysema may therefore have contributed to between-study variability and limited the generalizability of the findings. The small evidence base and insufficient subgroup data precluded separate analyses. The heterogeneity extended beyond differences in study populations and acquisition protocols. The included studies assessed different diaphragmatic parameters and comparator outcomes and reported their findings using incompatible formats, including group comparisons, correlations, and cut-off values. Therefore, sufficiently comparable effect estimates were unavailable for pooling, and a meta-analysis would not have produced a clinically meaningful or methodologically defensible summary estimate.

Furthermore, QUADAS-2 identified concerns regarding methodological quality, and the certainty of evidence was rated as very low using the GRADE approach. These findings emphasize that larger prospective studies employing standardized ultrasound protocols are required before DUS can be recommended for routine clinical use [2].

The clinical consequences of these limitations are substantial. The very low certainty of evidence and high or unclear risk of bias reduce confidence that the reported associations reflect reproducible effects rather than methodological or population-related differences. The absence of standardised acquisition protocols, measurement techniques, and reference values also prevents meaningful comparison of DUS measurements across studies, centres, ultrasound systems, and individual patients. Furthermore, because the available evidence is predominantly cross-sectional, it remains unknown whether DUS parameters change with disease progression, respond to treatment, or predict clinically relevant outcomes. Consequently, the current evidence does not support validated diagnostic thresholds, prognostic assessment, longitudinal monitoring, or DUS-guided treatment decisions in patients with F-ILD.

### 4.4. Strengths and Limitations

This review has several strengths, including a prospectively registered PROSPERO protocol, adherence to PRISMA guidelines, a comprehensive literature search, and independent study selection, data extraction, and quality assessment using validated tools.

Several limitations should also be acknowledged. Only six observational studies fulfilled the inclusion criteria, sample sizes were generally small, and methodological diversity precluded meta-analysis. Consequently, the findings should be interpreted cautiously and within the limitations of the available evidence.

The exclusion of non-English studies may have introduced language bias and resulted in the omission of relevant evidence.

QUADAS-2 was prespecified in the PROSPERO protocol and applied pragmatically to a body of evidence that primarily comprised cross-sectional association studies rather than conventional diagnostic accuracy studies. Furthermore, the included studies did not use a uniform reference standard for diaphragmatic dysfunction. The resulting risk-of-bias assessment should therefore be interpreted cautiously and should not be regarded as a formal evaluation of the diagnostic accuracy of DUS.

### 4.5. Future Perspectives

Future research should prioritize prospective multicenter studies using standardized DUS protocols, uniform reporting, and clinically relevant outcomes [2]. Establishing reproducible reference values and determining whether DUS adds value beyond PFT and HRCT are essential before clinical implementation. AI-based approaches also have shown preliminary potential in thoracic and diaphragmatic ultrasound, including automated detection and parameter measurement [25,26,27]. However, their applicability to F-ILD remains uncertain and requires prospective validation.

### 4.6. Conclusions

Current evidence suggests that DUS may detect diaphragmatic abnormalities in patients with F-ILD and may have future complementary value alongside established clinical assessments. However, the evidence is limited, heterogeneous, and of very low certainty and does not establish diagnostic accuracy, prognostic value, or usefulness for longitudinal monitoring. Accordingly, DUS cannot currently be recommended for routine clinical implementation in F-ILD, and prospective longitudinal validation is required.

## Data Availability

All data relevant to the study are included in the article or uploaded as Supplementary Information. Data can also be requested from the corresponding author. The study guarantors (SP, HZL and JRD) affirm that this manuscript is an honest, accurate and transparent account of the studies being reported; that no important aspects of the study have been omitted; and that any discrepancies from the study as planned (and, if relevant, registered) have been explained.

## Supplementary Materials

The following supporting information can be downloaded at: https://www.mdpi.com/article/10.3390/diagnostics16172759/s1, Supplementary S1: Deviations from protocol. Supplementary S2: PRISMA 2020 Checklist. Table S1: Database: mbase. Table S2: Database: Ovid MEDLINE. Table S3: Excluded references with reasons. Table S4: Overview of final judgement of each study based on QUADAS-2 sub-questions. Table S5: Detailed GRADE certainty-of-evidence assessment by outcome. Figure S1: Assessment of risk of bias. Figure S2: Concerns regarding applicability.

## Abbreviations

The following abbreviations are used in this manuscript:

6MWT	6-min walk test
BMI	body mass index
COPD	chronic obstructive pulmonary disease
CPFE	combined pulmonary fibrosis and emphysema
CTD-ILD	connective tissue disease-associated interstitial lung disease
DE	diaphragmatic excursion
DT	diaphragm thickness
DUS	diaphragmatic ultrasound
F-ILD	fibrosing interstitial lung disease
fHP	fibrotic hypersensitivity pneumonitis
fNSIP	fibrotic nonspecific interstitial pneumonia
IIP	idiopathic interstitial pneumonia
ILD	interstitial lung disease
IPF	idiopathic pulmonary fibrosis
kg/m^2^	kilograms per square metre
MRC	Medical Research Council
*n*	number of participants
NS	not specified
*p*	*p* value
PFT	pulmonary function testing
PPF	progressive pulmonary fibrosis
r	correlation coefficient
TF	thickening fraction
