# Supplementary material for: Diaphragmatic Ultrasound in Fibrosing Interstitial Lung Disease: A Systematic Review of Current Evidence
Source: Diagnostics (Basel). 2026 Aug 28;16(17):2759. doi: 10.3390/diagnostics16172759 (PMC13565281; doi:10.3390/diagnostics16172759)
Supplement: Supplementary file 1 [file diagnostics-16-02759-s001.zip › diagnostics-4478074-supplementary 2.0.pdf]

Supplementary S1:

Deviations from protocol.

1. Literature search:

Identification of new studies was carried out on 15th February 2025, apart from the original literature search that was initially conducted on 5th June 2023 and on 27th September 2023. This new search included full database searches and citation tracking (both forward and backward citation searching).

1. Forward and Backwards citation search.

We used Scopus to forward and backwards citation search.

2. Flow chart:

We used PRISMA guidelines recommend flow chart for updated systematic reviews.

4. Statistical analyses:

We planned to perform a meta-analysis initially. However, due to heterogeneity it was not feasible.

3. Date of final submission:

Dates were changed several times due to several reasons and PROSPERO has accepted the changes.

Table S1:

Database: mbase

#	Query	Results from 15 Feb 2025
1	exp interstitial lung disease/	162,450
2	exp lung fibrosis/	115,635
3	exp fibrosing alveolitis/	38,725
4	exp allergic pneumonitis/	14,994
5	exp interstitial pneumonia/	41,560
6	exp organizing pneumonia/	6,215
7	exp bronchiolitis obliterans organizing pneumonia/	2,481
8	exp asbestosis/	6,655
9	exp silicosis/	13,205
10	exp berylliosis/	1,376
11	exp lung sarcoidosis/	7,027
12	exp autoimmune lung disease/	2,858
13	exp connective tissue disease associated interstitial lung disease/	473
14	exp collagen vascular disease/	249
15	exp antisynthetase syndrome/	2,070
16	Interstitial lung disease*.mp.	47,755
17	Interstitial pulmonary disease*.mp.	512
18	Lung fibros*.mp.	55,542
19	Pulmonary fibros*.mp.	47,088
20	Fibrosing alveoliti*.mp.	39,212
21	Allergic pneumoniti*.mp.	8,438
22	asbestosi*.mp.	7,449
23	silicosi*.mp.	14,545
24	berylliosi*.mp.	1,490
25	interstitial pneumonia*.mp.	29,533
26	organizing pneumonia*.mp.	7,434
27	bronchiolitis obliterans organizing pneumonia*.mp.	2,985
28	Cryptogenic Organizing Pneumonia*.mp.	1,140
29	lung sarcoidosi*.mp.	7,080
30	Pulmonary sarcoidosi*.mp.	4,514
31	autoimmune lung disease*.mp.	166
32	autoimmune pulmonary disease*.mp.	4
33	connective tissue disease associated interstitial lung disease*.mp.	765
34	connective tissue disease associated interstitial pulmonary disease*.mp.	0
35	collagen vascular disease*.mp.	2,582
36	antisynthetase syndrome*.mp.	2,208

37	progressive-fibrosing interstitial lung disease*.mp.	408
38	progressive-fibrosing interstitial pulmonary disease*.mp.	0
39	Fibrotic Lung Disease*.mp.	1,781
40	Fibrotic Pulmonary Disease*.mp.	66
41	PF-ILD*.mp.	238
42	IPF*.mp.	22,172
43	PPF*.mp.	6,279
44	progressive pulmonary fibros*.mp.	787
45	progressive lung fibros*.mp.	173
46	fibrosing lung disease*.mp.	314
47	fibrosing pulmonary disease*.mp.	16
48	diffuse parenchymal lung disease*.mp.	877
49	diffuse parenchymal pulmonary disease*.mp.	3
50	idiopathic lung fibros*.mp.	171
51	idiopathic pulmonary fibros*.mp.	23,794
52	fibrotic idiopathic interstitial pneumonia*.mp.	55
53	pulmonary interstitial fibros*.mp.	364
54	lung interstitial fibros*.mp.	30
55	interstitial pulmonary fibros*.mp.	1,267
56	interstitial lung fibros*.mp.	293
57	Fibrotic Idiopathic Pulmonary Fibros*.mp.	3
58	Fibrotic Idiopathic Lung Fibros*.mp.	0
59	Desquamative interstitial pneumonia*.mp.	698
60	Acute interstitial pneumonia*.mp.	581
61	Hypersensitivity pneumoniti*.mp.	5,779
62	Idiopathic Interstitial Pneumoni*.mp.	2,701
63	Extrinsic Allergic Alveoliti*.mp.	1,085
64	CTD-ILD*.mp.	1,097
65	MCTD-ILD*.mp.	27
66	Scleroderma-ILD*.mp.	40
67	Rheumatoid arthritis-ILD*.mp.	34
68	RA-ILD*.mp.	1,058
69	Myositis-ILD*.mp.	51
70	Systemic sclerosis-ILD*.mp.	36
71	SSc-ILD*.mp.	1,563
72	Polymyositis-ILD*.mp.	2
73	Dermatomyositis-ILD*.mp.	17
74	SLE-ILD*.mp.	26
75	exp pneumoconiosis/	11,872

76	pneumoconios*.mp.	13,407
77	1 or 2 or 3 or 4 or 5 or 6 or 7 or 8 or 9 or 10 or 11 or 12 or 13 or 14 or 15 or 16 or 17 or 18 or 19 or 20 or 21 or 22 or 23 or 24 or 25 or 26 or 27 or 28 or 29 or 30 or 31 or 32 or 33 or 34 or 35 or 36 or 37 or 38 or 39 or 40 or 41 or 42 or 43 or 44 or 45 or 46 or 47 or 48 or 49 or 50 or 51 or 52 or 53 or 54 or 55 or 56 or 57 or 58 or 59 or 60 or 61 or 62 or 63 or 64 or 65 or 66 or 67 or 68 or 69 or 70 or 71 or 72 or 73 or 74 or 75 or 76	261,696
78	exp ultrasound/	258,705
79	echography/	418,100
80	echogram*.mp.	1,215
81	echograph*.mp.	494,772
82	echoscop*.mp.	228
83	echosound*.mp.	153
84	sonogram*.mp.	5,508
85	sonograph*.mp.	92,541
86	echotomograph*.mp.	1,660
87	ultraso*.mp.	913,612
88	78 or 79 or 80 or 81 or 82 or 83 or 84 or 85 or 86 or 87	1,136,373
89	77 and 88	7,060

Table S2:
Database: Ovid MEDLINE

#	Query	Results from 15 Feb 2025
1	exp Lung Diseases, Interstitial/	89,522
2	exp Pulmonary Fibrosis/	30,251
3	exp Idiopathic Pulmonary Fibrosis/	9,125
4	exp Idiopathic Interstitial Pneumonias/	1,672
5	exp Alveolitis, Extrinsic Allergic/	4,760
6	exp Pneumoconiosis/	21,428
7	exp Asbestosis/	4,508
8	exp Silicosis/	8,151
9	exp Berylliosis/	860
10	exp Sarcoidosis, Pulmonary/	3,636
11	exp organizing pneumonia/	1,418
12	exp Cryptogenic Organizing Pneumonia/	1,129
13	Pulmonary Fibros*.mp.	39,895
14	Lung Fibros*.mp.	6,426
15	Idiopathic Interstitial Pneumonia*.mp.	1,722
16	Pneumoconiosi*.mp.	8,633
17	Asbestosi*.mp.	5,231
18	Silicosi*.mp.	8,946
19	Berylliosi*.mp.	951
20	organizing pneumonia*.mp.	2,996
21	Cryptogenic Organizing Pneumonia*.mp.	1,448
22	bronchiolitis obliterans organizing pneumonia*.mp.	671
23	interstitial pneumonia*.mp.	10,186
24	Desquamative interstitial pneumonia*.mp.	423
25	Acute interstitial pneumonia*.mp.	383
26	allergic pneumoniti*.mp.	28
27	Hypersensitivity pneumoniti*.mp.	3,210
28	fibrosing alveoliti*.mp.	892
29	Extrinsic allergic alveoliti*.mp.	631
30	progressive-fibrosing interstitial lung disease*.mp.	216
31	progressive-fibrosing interstitial pulmonary disease*.mp.	0
32	Fibrotic Lung Disease*.mp.	996
33	Fibrotic Pulmonary Disease*.mp.	46
34	PF-ILD*.mp.	102
35	progressive pulmonary fibros*.mp.	444

36	progressive lung fibros*.mp.	92
37	Fibrotic Idiopathic Pulmonary Fibros*.mp.	1
38	Fibrotic Idiopathic lung Fibros*.mp.	0
39	fibrosing lung disease*.mp.	179
40	fibrosing pulmonary disease*.mp.	12
41	diffuse parenchymal lung disease*.mp.	480
42	diffuse parenchymal pulmonary disease*.mp.	1
43	idiopathic lung fibros*.mp.	83
44	idiopathic pulmonary fibros*.mp.	14,667
45	IPF*.mp.	11,396
46	Progressive pulmonary fibros*.mp.	444
47	PPF*.mp.	4,583
48	fibrotic idiopathic interstitial pneumonia*.mp.	38
49	pulmonary interstitial fibros*.mp.	269
50	lung interstitial fibros*.mp.	18
51	interstitial pulmonary fibros*.mp.	777
52	interstitial lung fibros*.mp.	161
53	Interstitial Lung Disease*.mp.	19,340
54	Interstitial Pulmonary Disease*.mp.	325
55	autoimmune lung disease*.mp.	30
56	autoimmune pulmonary disease*.mp.	3
57	connective tissue disease associated interstitial lung disease*.mp.	263
58	connective tissue disease associated interstitial pulmonary disease*.mp.	0
59	CTD-ILD*.mp.	464
60	MCTD-ILD*.mp.	7
61	Rheumatoid arthritis-ILD*.mp.	8
62	RA-ILD*.mp.	522
63	Scleroderma-ILD*.mp.	8
64	Myositis-ILD*.mp.	19
65	Systemic sclerosis-ILD*.mp.	11
66	SSc-ILD*.mp.	644
67	Polymyositis-ILD*.mp.	0
68	Dermatomyositis-ILD*.mp.	4
69	SLE-ILD*.mp.	8
70	collagen vascular disease*.mp.	1,511
71	antisynthetase syndrome*.mp.	785
72	Pulmonary Sarcoidosi*.mp.	2,785
73	Lung Sarcoidosi*.mp.	115
74	1 or 2 or 3 or 4 or 5 or 6 or 7 or 8 or 9 or 10 or 11 or 12 or 13 or 14 or 15 or 16 or 17 or 18 or 19 or 20 or 21 or 22 or 23 or 24 or 25 or 26 or 27 or 28 or 29 or 30 or 31 or 32 or 33 or 34 or 35 or 36 or 37 or 38 or 39 or 40 or 41 or 42 or 43 or 44 or 45 or 46 or 47 or 48 or 49 or 50 or 51 or 52 or 53 or 54 or 55 or 56 or 57 or 58 or	124,038

	59 or 60 or 61 or 62 or 63 or 64 or 65 or 66 or 67 or 68 or 69 or 70 or 71 or 72 or 73	
75	exp Ultrasonography/	506,559
76	echocardiograph*.mp.	234,951
77	echogram*.mp.	809
78	echograph*.mp.	11,452
79	echoscop*.mp.	162
80	echosound*.mp.	147
81	sonogram*.mp.	3,975
82	sonograph*.mp.	63,730
83	echotomograph*.mp.	790
84	ultraso*.mp.	633,349
85	75 or 76 or 77 or 78 or 79 or 80 or 81 or 82 or 83 or 84	874,518
86	74 and 85	2,394

Table S3: Excluded references with reasons.

No.	First author/år	References	Reason for exclusion
1.	Flores 2023	DIAGNOSTIC ACCURACY OF LUNG ULTRASOUND FOR DETECTING INTERSTITIAL LUNG DISEASE AMONG PATIENTS WITH SYSTEMIC SCLEROSIS: A COMPARATIVE STUDY OF TWO SCANNING PROTOCOLS.	Conferance abstract
2.	Shchepikhin 2023	Possibilities of non-invasive diagnosis of fibrotic phenotype of interstitial lung diseases	Wrong intervention
3.	Saeed 2023	Study of diaphragmatic mobility by chest ultrasound and changes in maximal respiratory pressures in patients with interstitial pulmonary fibrosis before and after pulmonary rehabilitation	Wrong patient population
4.	Ahunova 2023	PULMONARY ULTRASOUND: DIAGNOSTIC POSSIBILITIES FOR INTERSTITIAL LUNG DISEASE ASSOCIATED WITH RHEUMATOID ARTHRITIS	Review
5.	Machiridza 2022	Advanced pulmonary fibrosis as an extra-intestinal manifestation of inflammatory bowel disease	Case report
6.	Koshy 2023	Descriptive study of the role of ultrasound in the evaluation of patients with interstitial lung disease associated with autoimmune connective tissue disorders	Wrong patient population
7.	Boccatonda 2023	All B-lines are equal, but some B-lines are more equal than others	Review
8.	Joerg 2022	Chest ultrasound findings in usual interstitial pneumonia patterns: a pilot study	Excluded due to incomparable inclusion criteria

9.	Wang 2022	Serum B-cell activating factor and lung ultrasound B-lines in connective tissue disease related interstitial lung disease	Wrong intervention
10.	Gutierrez 2022	Ultrasound may detect subclinical interstitial lung diseasein systemic sclerosis	Conferance abstract
11.	GomesGuerra 2023	The Role of Lung Ultrasound in Systemic Sclerosis: A Systematic Review	Review
12.	Vicente-Rabaneda 2022	Response to "Subclinical Interstitial Lung Disease in Patients with Systemic Sclerosis. A Pilot Study on the Role of Ultrasound"	Letter to the editor
13.	Zhang 2023	A study of the diagnostic value of a modified transthoracic lung ultrasound scoring method in interstitial lung disease	Wrong patient population
14.	Ovsyannikova 2023	Specificity and sensitivity of ultrasound lung in patients with rheumatic diseases	Conferance abstract
15.	Ho 2023	US evaluation of ILD in autoimmune disease	Conferance abstract
16.	Ovsyannikova 2023	The role of ultrasound lung artifacts in the diagnosis of interstitial lung disease in patients with rheumatic diseases	Wrong study design
17.	Ovsyannikova 2023	Echographic examination of the lung as a method to assess interstitial lung disease of systemic sclerosis	Conferance abstract
18.	Khoury 2022	Is all that fibrosis idiopathic? Usual interstitial pneumonia in IgG4 related disease	Case report
19.	Saeed 2022	Correlation between diaphragmatic mobility by transthoracic ultrasound and echocardiography findings in patients with idiopathic pulmonary fibrosis	Wrong intervention
20.	Watanabe 2022	Lung Ultrasound in Patients with Connective Tissue Disease-associated Interstitial Lung Disease; Utility of Single Evaluation in Clinical Practice	Conferance abstract
21.	Fairchild 2020	Development and Assessment of a Novel Lung Ultrasound Interpretation Criteria for the Detection of Interstitial Lung Disease in Systemic Sclerosis	Wrong patient population

22.	Wang 2019	Lung ultrasound B-lines and serum KL-6 correlate with the severity of idiopathic inflammatory myositis-associated interstitial lung disease	Wrong intervention
23.	Manolescu 2019	Ultrasound mapping of lung changes in idiopathic pulmonary fibrosis	Duplicate
24.	Huang 2022	Evaluation of connective tissue disease-related interstitial lung disease using ultrasound elastography: a preliminary study	Wrong patient population
25.	Huang 2023	Screening value of lung ultrasound in connective tissue disease related interstitial lung disease	Wrong patient population
26.	Bruni 2022	Lung Ultrasound B-Lines in the Evaluation of the Extent of Interstitial Lung Disease in Systemic Sclerosis	Wrong patient population
27.	Gargani 2022	Lung ultrasound B-lines in systemic sclerosis: cut-off values and methodological indications for interstitial lung disease screening	Wrong patient population
28.	Gutierrez 2022	The emerging role of ultrasound in detecting interstitial lung disease in patients with rheumatoid arthritis	Wrong setting
29.	DiCarlo 2022	Lung ultrasound in patients with rheumatoid arthritis: definition of significant interstitial lung disease	Wrong patient population
30.	Soldati 2022	Operative Use of Thoracic Ultrasound in Respiratory Medicine: A Clinical Study	Wrong indication
31.	Pitsidianakis 2022	Performance of Lung Ultrasound for Monitoring Interstitial Lung Disease	Excluded due to incomparable inclusion criteria
32.	OVSYNNIKOVA 2022	SENSITIVITY AND SPECIFICITY OF ULTRASONOGRAPHY FOR DIAGNOSIS OF INTERSTITIAL LUNG DISEASE IN PATIENTS WITH RHEUMATIC DISEASES	Conferance abstract
33.	D'Orazio 2022	TRANSTHORACIC LUNG SONOGRAPHY (LUS)-USEFUL SURVEILLANCE TOOL in SYSTEMIC SCLEROSIS ASSOCIATED INTERSTITIAL LUNG DISEASE?	Conferance abstract

34.	RezaBeigi 2022	LUNG ULTRASOUND for INTERSTITIAL LUNG DISEASE DETECTION in A COHORT of SYSTEMIC SCLEROSIS PATIENTS: ROLE of B-LINE and PLEURAL LINE MODIFICATIONS	Conferance abstract
35.	Ovsyannikova 2022	SPECIFICITY and SENSITIVITY of LUNG ULTRASOUND (LUS) in RHEUMATIC DISEASES on RUSSIAN COHORTS of PATIENTS	Conferance abstract
36.	Ovsyannikova 2022	USE of ULTRASOUND to DIAGNOSE INTERSTITIAL LUNG DISEASE in RHEUMATIC DISEASES on RUSSIAN COHORTS of PATIENTS	Conferance abstract
37.	Brigante 2022	SCREENING of INTERSTITIAL LUNG DISEASE in PATIENTS with RHEUMATOID ARTHRITIS THROUGH AN ULTRASONOGRAPHY PROTOCOL	Conferance abstract
38.	Gutierrez 2022	ULTRASOUND MAY DETECT SUBCLINICAL INTERSTITIAL LUNG DISEASE in SYSTEMIC SCLEROSIS	Conferance abstract
39.	Gutierrez 2022	ULTRASOUND AS A POTENTIAL TOOL to DETECT INTERSTITIAL LUNG DISEASE in DAILY CLINICAL SETTING of PATIENTS with RHEUMATOID ARTHRITIS	Conferance abstract
40.	Greco 2022	Diaphragmatic ultrasound evaluation among scleroderma patients with interstitial lung disease	Only Abstract
41.	Rao 2021	Imaging in connective tissue disease-associated interstitial lung disease	Review
42.	Lux 2021	Diaphragm Ultrasound in the Evaluation of Diaphragmatic Dysfunction in Lung Disease	Wrong study design
43.	Ruaro 2021	High-resolution computed tomography and lung ultrasound in patients with systemic sclerosis: Which one to choose?	Review
44.	Lisha 2021	Diagnostic validity of lung ultrasonogram in comparison with high-resolution computed tomography in interstitial lung disease associated with connective tissue disease	Wrong patient population

45.	Zhou 2021	Lung mass density prediction using machine learning based on ultrasound surface wave elastography and pulmonary function testing	Wrong study design
46.	Yan 2021	Utility of lung ultrasound to identify interstitial lung disease: An observational study based on the	Wrong patient population
		STROBE guidelines	
47.	Wang 2021	The role of lung ultrasound B-lines and serum KL-6 in the screening and follow-up of rheumatoid arthritis patients for an identification of interstitial lung disease: review of the literature, proposal for a preliminary algorithm, and clinical application	Wrong patient population
48.	Zhu 2021	Risk assessment in interstitial lung disease: the incremental prognostic value of cardiopulmonary ultrasound	Wrong setting
49.	Fotoh 2021	Serum Krebs von den Lungen-6 and lung ultrasound B lines as potential diagnostic and prognostic factors for rheumatoid arthritis-associated interstitial lung disease	Wrong intervention
50.	Buda 2021	Clinical impact of vertical artifacts changing with frequency in lung ultrasound	Wrong patient population
51.	Buda 2021	Lung ultrasound in the screening of pulmonary interstitial involvement secondary to systemic connective tissue disease: A prospective pilot study involving 180 patients	Wrong patient population
52.	Reyes-Long 2021	Subclinical Interstitial Lung Disease in Patients with Systemic Sclerosis. A Pilot Study on the Role of Ultrasound	Wrong patient population
53.	Kumar 2021	Performance of semi-quantitative lung ultrasound in the assessment of disease severity in interstitial lung disease	Wrong setting
54.	Legue 2021	ThOracic Ultrasound in Idiopathic Pulmonary Fibrosis Evolution (TOUPIE): Research protocol of a multicentric prospective study	Protocol

55.	Fairchild 2021	Development and Assessment of Novel Lung Ultrasound Interpretation Criteria for the Detection of Interstitial Lung Disease in Systemic Sclerosis	Duplicate
56.	Mena-Vazquez 2021	Utility of pulmonary ultrasound to identify interstitial lung disease in patients with rheumatoid arthritis	Wrong patient population
57.	Vasarmidi 2021	A prospective evaluation of lung ultrasonography in patients with fibrotic interstitial lung diseases	Conferance abstract
58.	Bernardinello 2021	Diaphragm ultrasound in patients with interstitial lung disease (ILD): a prospective study	Conferance abstract
59.	Bruni 2021	Lung ultrasound to assess the severity of interstitial lung disease in systemic sclerosis	Conferance abstract
60.	Wang 2020	Lung ultrasound B-lines and serum KL-6 correlate with the severity of idiopathic inflammatory myositis-associated interstitial lung disease	Wrong intervention
61.	Mento 2020	Quantitative Lung Ultrasound Spectroscopy Applied to the Diagnosis of Pulmonary Fibrosis: The First Clinical Study	Wrong patient population
62.	Gargani 2020	Prognostic Value of Lung Ultrasound B-Lines in Systemic Sclerosis	Wrong patient population
63.	Volpicelli 2020	Lung Ultrasound B-Lines in Interstitial Lung Disease: Moving From Diagnosis to Prognostic Stratification	Letter to the editor
64.	Krome 2020	Thoracic ultrasound in systemic sclerosis	Letter to the editor
65.	Srivastava 2020	Transthoracic ultrasonography in patients with interstitial lung disease	Excluded due to incomparable inclusion criteria
66.	Comes 2020	Early diagnosis of idiopathic pulmonary fibrosis: Closer to the goal?	Letter to the editor
67.	SerraltaSanMartin 2020	Clinical ultrasonography in systemic autoimmune diseases	Wrong indication
68.	Gasperini 2020	The predictive role of lung ultrasound in progression of scleroderma interstitial lung disease	Wrong patient population

69.	Abdullayeva 2020	Lung ultrasound and ultrasound elastography for diagnosis and followup of interstitial lung diseases	Conferance abstract
70.	Maci 2020	The role of Transthoracic Ultrasound in the study of interstitial lung disease: HRCT versus ultrasound pattern	Conferance abstract
71.	Chawla 2020	Utility of Lung Ultrasound in Interstitial Lung Disease - A Paradigm Shift	Conferance abstract
72.	DICarlo 2020	Lung ultrasound in patients with rheumatoid arthritis and the definition of significant interstitial lung disease	Conferance abstract
73.	Godoy-Navarrete 2020	Lung ultrasound utility in interstitial lung disease detection in rheumatoid arthritis	Conferance abstract
74.	Kirillova 2020	Lung ultrasound in patients with secondary interstitial lung diseases	Conferance abstract
75.	Buda 2019	Utility of lung ultrasound in ANCA-associated vasculitis with lung involvement	Wrong patient population
76.	Man 2019	Correlation between transthoracic lung ultrasound score and HRCT features in patients with interstitial lung diseases	Excluded due to incomparable inclusion criteria
77.	Hassan 2019	Lung Ultrasound as a Screening Method for Interstitial Lung Disease in Patients with Systemic Sclerosis	Wrong patient population
78.	Zhang 2019	A quantitative method for measuring the changes of lung surface wave speed for assessing disease progression of interstitial lung disease	Wrong patient population
79.	WanguemertPerez 2019	Ultrasound Application With Acoustic Structure Quantification (ASQ) in Interstitial Lung Diseases	Letter to the editor
80.	Verschakelen 2019	Elastography of the lung using US: A noninvasive, reproducible tool to detect and stage interstitial lung disease	Letter to the editor
81.	Dogan 2019	Ultrasonographic evaluation of lung parenchyma involvement in sarcoidosis	Wrong setting
82.	Zhou 2019	Lung US surface wave elastography in interstitial lung disease staging	Excluded due to incomparable inclusion criteria
83.	Zhang 2019	Lung Ultrasound Surface Wave Elastography for Assessing Interstitial Lung Disease	Wrong intervention

84.	Fairchild 2019	Development and preliminary validation of a novel lung ultrasound interpretation criteria for the detection of interstitial lung disease in Patients with Systemic Sclerosis	Conferance abstract
85.	Smargiassi 2019	Possible role of chest ultrasonography for the evaluation of peripheral fibrotic changes in patients affected by IPF	Conferance abstract
86.	Man 2019	The role of lung ultrasonography in the assessment of idiopathic pulmonary fibrosis -correlation with HRCT finding	Conferance abstract
87.	Manolescu 2019	Twelve lung zones protocol of ultrasound mapping in idiopathic pulmonary fibrosis	Conferance abstract
88.	Roganovic 2019	Correlation of the findings of ultrasound examination of the lungs and interstitial lung fibrosis in patients with systemic sclerosis	Conferance abstract
89.	Masiak 2019	Clinical usefulness of lung ultrasound in active granulomatosis with polyangiitis with lung involvement-preliminary data	Conferance abstract
90.	Ferro 2019	Lung ultrasound of pleural irregularities in sub-clinical primary sjogren's syndromelung involvement: A single centre experience	Conferance abstract
91.	Gutierrez 2019	Diagnostic value of pulmonary ultrasound in the assessment of interstitial lung disease in systemic sclerosis	Conferance abstract
92.	Mattioli 2018	Point-of-care thoracic ultrasound for the detection of interstitial lung disease in scleroderma patients: A preliminary report	Conferance abstract
93.	Asano 2018	Validity of Ultrasound Lung Comets for Assessment of the Severity of Interstitial Pneumonia	Excluded due to incomparable inclusion criteria
94.	Petruzzelli 2018	Ultrasound B-lines for detection of late lung fibrosis in breast cancer patients after radiation therapy	Wrong outcomes
95.	Fehr 2018	Transthoracic ultrasound in the detection of interstitial pulmonary fibrosis in patients with rheumatic connective tissue diseases	Wrong patient population
96.	Singh 2018	The Use of M-Mode Ultrasonography to Differentiate the Causes of B Lines	Wrong patient population

97.	Boccatonda 2018	Ultrasound evaluation of diaphragmatic mobility in patients with idiopathic lung fibrosis: A pilot study	Duplicate
98.	Marchetti 2018	New insights in the use of pleural ultrasonography for diagnosis and treatment of pleural disease	Review
99.	Manolescu 2018	The reliability of lung ultrasound in assessment of idiopathic pulmonary fibrosis	Wrong study design
100.	Tardella 2018	Ultrasound B-lines in the evaluation of interstitial lung disease in patients with systemic sclerosis	Wrong patient population
101.	Ferro 2018	Ultrasound of pleural irregularities: A promising tool in primary Sjogren's syndrome (pSS)-associated interstitial lung disease (ILD). a functional and CT correlation study	Only Abstract
102.	Rotondo 2018	Lung ultrasound in one-year follow-up of a SSc patient cohort: It could be the onset of abnormal ultrasound signs an important imaging tool to reduce the frequency of chest tomographic assessment?	Conferance abstract
103.	Tinazzi 2018	Lung ultrasonography: Possible role as predictor of pulmonary involvement in patients affected by systemic sclerosis complicated by either interstitial lung disease or pulmonary arterial hypertension	Conferance abstract
104.	Dogan 2018	Ultrasonographic evaluation of lung parenchyma in sarcoidosis	Conferance abstract
105.	Pitsidianakis 2018	A prospective evaluation of lung ultrasonography in patients with fibrotic interstitial lung diseases; Preliminary results	Conferance abstract
106.	Roganovic 2018	The role of ultrasound in the discovery of interstitial changes in the lungs in patients with systemic connective tissue disease	Conferance abstract
107.	Cioffi 2018	Interstitial lung disease in idiopathic inflammatory myopathies: Ultrasound assessment of pleural irregularities and comparison with high resolution computed tomography	Conferance abstract

108.	Gutierrez 2018	Ultrasound diagnostic and predictive value of interstitial lung disease in systemic sclerosis. diagnostic and predictive value of ultrasound in the assessment of interstitial lung disease	Conferance abstract
109.	Moreno 2018	Pulmonary ultrasound in the assessment of interstitial lung disease in rheumatoid arthritis	Conferance abstract
110.	Barbieri 2018	Effectiveness of chest us (ultrasound) in the diagnosis of ild (interstitial lung diseases) in an unselected population: A prospective study	Conferance abstract
111.	Oliveira 2018	Lung ultrasonography in systemic sclerosis	Conferance abstract
112.	Tardella 2018	Ultrasound B-lines in the evaluation of interstitial lung disease in patients with systemic sclerosis: Cut-off point definition for the presence of significant pulmonary fibrosis	Duplicate
113.	Zhang 2018	An Ultrasound Surface Wave Technique for Assessing Skin and Lung Diseases	Wrong study design
114.	Zhang 2017	Lung Ultrasound Surface Wave Elastography: A Pilot Clinical Study	Wrong patient population
115.	Smargiassi 2017	Chest ultrasonography in health surveillance of asbestos-related lung diseases	Wrong patient population
116.	Dubinsky 2017	Correlation of B-Lines on Ultrasonography With Interstitial Lung Disease on Chest Radiography and CT Imaging	Wrong study design
117.	Zhu 2017	Integrative Cardiopulmonary Ultrasound for Interstitial Lung Disease Assessment: Correlation between Lung Ultrasound Performance and Cardiac Involvement	Wrong intervention
118.	Sperandeo 2017	Lung Ultrasound and Chest X-Rays: Together to Improve the Diagnosis	Letter to the editor
119.	Tarnoki 2017	Ultrasound assessment of diaphragmatic motion in patients with interstitial lung disease	Only Abstract
120.	Buda 2017	Lung ultrasound in the evaluation of interstitial lung diseases in the course of connective tissue diseases	Wrong study design
121.	Cui 2017	Ultrasonographic features of typical HRCT signs of interstitial lung diseases	Conferance abstract

122.	Tardella 2017	Ultrasound b-lines in the evaluation of interstitial lung disease in patients with systemic sclerosis: cut-off point definition for the presence of pulmonary fibrosis	Conferance abstract
123.	Khor 2017	Bubble artefacts on lung ultrasound are associated with radiological honeycombing	Conferance abstract
124.	Ferro 2017	Correlation between lung ultrasound, HRCT findings and pulmonary function tests in primary sjogren's syndrome (PSS)-associated interstitial lung disease	Conferance abstract
125.	Osborn 2017	A non-invasive ultrasound surface wave elastography technique for assessing interstitial lung disease	Conferance abstract
126.	Kalra 2017	Lung ultrasound surface wave elastography-Preliminary measurements in patients with interstitial lung diseases	Conferance abstract
127.	Vassalou 2017	Modified Lung Ultrasonographic Technique for Evaluation of Idiopathic Pulmonary Fibrosis: Lateral Decubitus Position	Study population is included in a nother selected study
128.	Sambataro 2017	Is there any role for thoracic ultrasound for interstitial lung disease underlying rheumatologic conditions?	Review
129.	Vasco 2017	Assessment of interstitial lung disease in Sjogren's syndrome by lung ultrasound: a pilot study of correlation with high-resolution chest tomography	Wrong patient population
130.	Minthara 2016	Acoustic radiation force impulse elastography based on elasticity imaging for early detection of idiopathic pulmonary fibrosis	Conferance abstract
131.	Vizioli 2017	Integrated Use of Lung Ultrasound and Chest X-Ray in the Detection of Interstitial Lung Disease	Wrong patient population
132.	Gutierrez 2016	Ultrasound in the interstitial pulmonary fibrosis. Can it facilitate a best routine assessment in rheumatic disorders?	Wrong study design
133.	Agmy 2016	Assessment of transthoracic sonography in patients with interstitial lung diseases	Conferance abstract

134.	Rotondo 2016	Chest ultrasound signs of interstitial lung disease in systemic sclerosis patients: A comparison between high resolution chest computed tomography findings	Conferance abstract
135.	Petruzzelli 2016	The Potential Role of Lung Ultrasound B-Lines for Detection of Lung Radio-Induced Toxicity in Breast Cancer Patients after Radiation Therapy	Wrong patient population
136.	Baston 2016	Lung ultrasound in acute respiratory distress syndrome and beyond	Wrong study design
137.	Sperandeo 2016	Chest Ultrasonography as a Screening Tool for High-Resolution Computed Tomography Referral in Patients With Systemic Sclerosis-A Future Perspective: Comment on the Article by Suliman et al	Letter to the editor
138.	Sheveleva 2016	DIAPHRAGM KINETICS IN PULMONARY FIBROSIS PATIENTS AND GASTROESOPHAGEAL REFLUX	Wrong intervention
139.	Yasukawa 2016	Differential Diagnosis of Interstitial Pneumonia by Home Ultrasound Examination	Conferance abstract
140.	Buda 2016	Lung Ultrasonography in the Evaluation of Interstitial Lung Disease in Systemic Connective Tissue Diseases: Criteria and Severity of Pulmonary Fibrosis - Analysis of 52 Patients	Wrong setting
141.	Santana 2016	Identifying decreased diaphragmatic mobility and diaphragm thickening in interstitial lung disease: the utility of ultrasound imaging	Wrong setting
142.	Gigante 2016	Lung ultrasound in systemic sclerosis: correlation with high-resolution computed tomography, pulmonary function tests and clinical variables of disease	Wrong patient population
143.	Abdulaziz 2015	Rheumatoid arthritis-associated interstitial lung disease Topical Collection on Interstitial Lung Disease	Review
144.	Sperandeo 2015	Ultrasound signs of pulmonary fibrosis in systemic sclerosis as timely indicators for chest computed tomography	Excluded due to incomparable inclusion criteria

145.	Rea 2015	A farewell to B-lines: Ageing and disappearance of ultrasound artifacts as a diagnostic tool	Letter to the edito
146.	Trovato 2015	The resistible rise of B-line lung ultrasound artefacts	Letter to the editor
147.	Karalilova 2015	The correlation of high frequency ultrasound of dermal thickness and lung ultrasound for pulmonary involvement in early diffuse systemic sclerosis	Conferance abstract
148.	Singh 2015	M-mode ultrasound to define pleural and subpleural morphology among patients with interstitial lung syndromes	Conferance abstract
149.	Comert 2015	Ultrasound in the assessment of interstitial lung diseases: Correlation with highresolution computed tomography and lung functions	Conferance abstract
150.	Asano 2015	Validity of the ultrasound lung comets signs for assessment of severity of interstitial pneumonia	Conferance abstract
151.	Pinal-Fernandez 2015	Pleural irregularity, a new ultrasound sign for the study of interstitial lung disease in systemic sclerosis and antisynthetase syndrome	Wrong patient population
152.	Moazedi-Fuerst 2015	Sonographic assessment of interstitial lung disease in patients with rheumatoid arthritis, systemic sclerosis and systemic lupus erythematosus	Wrong patient population
153.	Moazedi-Fuerst 2014	Ultrasound screening for interstitial lung disease in rheumatoid arthritis	Excluded due to incomparable inclusion criteria
154.	Fernandez 2014	Correlation of ultrasound B-lines with high-resolution computed tomography in antisynthetase syndrome	Wrong patient population
155.	Reissig 2014	Lung ultrasound in community-acquired pneumonia and in interstitial lung diseases	Case report
156.	Hasan 2014	B-lines: Transthoracic chest ultrasound signs useful in assessment of interstitial lung diseases	Excluded due to incomparable inclusion criteria
157.	Cogliati 2014	Standard and pocket-Size lung ultrasound devices can detect interstitial lung disease in rheumatoid arthritis patients	Wrong patient population

158.	Trovato 2014	Thoracic ultrasound: Possible complementary criteria for the assessment of pulmonary fibrosis	Letter to the editor
159.	Mohammadi 2014	Comparison of a new, modified lung ultrasonography technique with high-resolution CT in the diagnosis of the alveolo-interstitial syndrome of systemic scleroderma	Wrong patient population
160.	Santana 2014	Diaphragmatic dysfunction in interstitial lung disease: An ultrasonography study	Only Abstract
161.	LopezMartinez 2014	Lung ultrasound for detecting interstitial lung disease in patients with systemic sclerosis	Only Abstract
162.	Antivalle 2014	Lung ultrasound screening for interstitial lung disease in rheumatoid arthritis comparison with usual detection algorithms in clinical practice	Conferance abstract
163.	Buda 2014	Transthoracic ultrasonography (TUS) in the diagnosis of pulmonary fibrosis in patients diagnosed with systemic connective tissue disease	Conferance abstract
164.	Buda 2014	The severity of pulmonary fibrosis in transthoracic ultrasonography (TUS)	Conferance abstract
165.	Gutierrez 2014	Validity of ultrasound in the assessment of pulmonary fibrosis in systemic sclerosis: Correlation with high resolution computed tomography, pulmonary function test and histological findings	Conferance abstract
166.	Karalilova 2014	Lung ultrasound for evaluation of pulmofibrosis in systemic sclerosis	Conferance abstract
167.	Sperandeo 2014	Lung ultrasound imaging usefulness for assessing the severity of pulmonary fibrosis in systemic sclerosis. Relationship with high-resolution computed tomography and nailfold videocapillaroscopy	Only Abstract
168.	Barskova 2013	Lung ultrasound for the screening of interstitial lung disease in very early systemic sclerosis	Wrong patient population
169.	Hasan 2013	Comet tail artifact: A transthoracic chest ultrasound sign useful in assessment of interstitial lung diseases	Conferance abstract

170.	Gargani 2013	Ultrasound in lung fibrosis	Conferance abstract
171.	Torzillo 2013	Lung ultrasound for the diagnosis of interstitial disease in rheumatoid arthritis	Conferance abstract
172.	Gargani 2013	Lung ultrasound for the screening of interstitial lung disease in very early systemic sclerosis	Conferance abstract
173.	Crisafulli 2013	Pulmonary fibrosis and lung ultrasound	Only Abstract
174.	Tardella 2012	Ultrasound in the assessment of pulmonary fibrosis in connective tissue disorders: Correlation with high-resolution computed tomography	Excluded due to incomparable inclusion criteria
175.	Picano 2012	Ultrasound lung comets: The shape of lung water	Letter to the editor
176.	DelleSedie 2012	The promising role of lung ultrasound in systemic sclerosis	Review
177.	Sperandeo 2012	Transthoracic ultrasound in patients with systemic sclerosis	Conferance abstract
178.	Sperandeo 2012	Role of Transthoracic ultrasound in the study of pulmonary fibrosis in patients with Systemic Sclerosis	Conferance abstract
179.	Barskova 2012	Lung ultrasound for the screening of interstitial lung disease in SSc	Conferance abstract
180.	Reissig 2012	Pneumonia and diffuse parenchymal lung diseases in lung ultrasound	Review
181.	Gutierrez 2011	Utility of a simplified ultrasound assessment to assess interstitial pulmonary fibrosis in connective tissue disorders - preliminary results	Excluded due to incomparable inclusion criteria
182.	Petrova 2011	Features of pulmonary involvement in patients with diffuse connective tissue diseases	Conferance abstract
183.	Kielhauser 2011	Ultrasound screening for intestinal lung disease in rheumatoid arthritis	Only Abstract
184.	Oargani 2011	Ultrasound lung comets in systemic sclerosis: A chest sonography hallmark of pulmonary interstitial fibrosis	Duplicate
185.	Doveri 2011	Ultrasound lung comets as a long-term prognostic determinant in systemic sclerosis	Conferance abstract

186.	Gargani 2009	Ultrasound lung comets in systemic sclerosis: a chest sonography hallmark of pulmonary interstitial fibrosis	Excluded due to incomparable inclusion criteria
187.	Oargani 2010	Ultrasound lung comets in systemic sclerosis: A chest sonography hallmark of pulmonary interstitial fibrosis	Duplicate
188.	Gutierrez 2010	Ultrasound lung comets. Validity, reproducibility and feasibility of a simplified assessment of pulmonary interstitial fibrosis in connective tissue disorders	Conferance abstract
189.	Capati 2010	Ultrasound lung comets as a long-term prognostic determinant in systemic sclerosis	Conferance abstract
190.	Copetti 2010	Ultrasound Pattern in Pulmonary Fibrosis: Have the Vertical Artifacts Disappeared?	Letter to the editor
191.	Sedie 2010	Ultrasound lung comets in systemic sclerosis: A useful tool to detect lung	Letter to the editor
		interstitial fibrosis	
192.	Gargani 2009	Ultrasound lung comets as a long-term prognostic determinant in systemic sclerosis	Conferance abstract
193.	Volpicelli 2007	Significance of comet tail artifacts at lung ultrasound	Letter to the editor
194.	SerranoRamos 2008	Sun rays diaphragm: An ultrasonographic sign of interstitial involvement in the base of the lung	Review
195.	Doveri 2008	Ultrasound lung comets: New echographic sign of lung interstitial fibrosis in systemic sclerosis	Wrong outcomes
196.	Soldati 2006	Sonographic findings in pulmonary diseases	Wrong setting
197.	Volpicelli 2006	Bedside lung ultrasound in the assessment of alveolar-interstitial syndrome	Wrong outcomes
198.	Reissig 2003	Transthoracic sonography of diffuse parenchymal lung disease: The role of comet tail artifacts	Wrong setting
199.	Strunk 2001	Sonographic incidental finding of a lymphangioleiomyomatosis (LAM)	Case report

200.	Lichtenstein 1997	The comet-tail artifact: An ultrasound sign of alveolar-interstitial syndrome	Wrong patient population
201.	Giron 1994	Pleural plaques and thoracic imaging	Letter to the editor
202.	Burilkov 1983	Ultrasonic diagnosis of pleural changes in asbestosis	Case report
203.	Ross 1968	Ultrasonic examination of the lung	Wrong setting
204.	Viikeri 1968	Ultrasonic examination of pleural thickenings and calcifications in occupational asbestosis	Wrong patient population
205.	Vicente-Rabaneda 2022	Ultrasound evaluation of interstitial lung disease in rheumatoid arthritis and autoimmune diseases.	Review
206.	Vicente-Rabaneda 2021	Response to "Subclinical Interstitial Lung Disease in Patients with Systemic Sclerosis. A Pilot Study on the Role of Ultrasound".	Letter to the editor
207.	Wang 2020	[Transthoracic ultrasonographic features of typical high-resolution computed tomography signs of interstitial lung diseases]	Excluded due to incomparable inclusion criteria
208.	Sambataro 2017	Is there any role for thoracic ultrasound for interstitial lung disease underlying rheumatologic conditions? Reply.	Letter to the editor
209.	Zhang 2016	A noninvasive ultrasound elastography technique for measuring surface waves on the lung.	Wrong intervention
210.	Raso 2015	A soft computing-based B-line analysis for objective classification of severity of pulmonary edema and fibrosis.	Letter to the editor.
211.	Laria 2015	Ultrasound in Rheumatologic Interstitial Lung Disease: A Case Report of Nonspecific Interstitial Pneumonia in Rheumatoid Arthritis.	Case report
212.	Steen 2005	The lung in systemic sclerosis.	Wrong intervention
213.	Sandron 1993	[Ultrasonographic aspect of asbestos infracostal pleural plates].	Wrong patient population
214.	Sample 1977	Ultrasound and computed tomography of the pleura.	Letter to the editor

215.	ROLFO 1964	[EXPERIMENTAL AND CLINICAL RESEARCH ON THE POSSIBILITIES OF THE USE OF ULTRASONICS IN THE DIAGNOSIS OF SOME CHEST DISEASES].	Article is not available
216.	HADJIOLOFF 1963	[ULTRASONIC TREATMENT OF SILICOSIS]	Review
217.	Sperandeo 2017	Lung Ultrasound and Chest X-Rays: Together to Improve the Diagnosis...Vizioli, L, Ciccarese, F, Forti, P et al. Integrated use of lung ultrasound and chest X-ray in the detection of interstitial lung disease. Respiration 2017; 93:15-22	Letter to the editor
218.	ÇAKIREDİS 2016	Effectiveness of Thoracic Ultrasonography in the Evaluation of the Severity of Pulmonary Involvement in Patients With Systemic Sclerosis.	Wrong patient population
219.	Vizioli 2016	Integrated Use of Lung Ultrasound and Chest X-Ray in the Detection of Interstitial Lung Disease.	Duplicate
220.	Sperandeo 2016	Chest Ultrasonography as a Screening Tool for High-Resolution Computed Tomography Referral in Patients With	Letter to the editor
		Systemic Sclerosis-A Future Perspective: Comment on the Article by Suliman et al...Suliman YA, Dobrota R, Huscher D, et al. Pulmonary function test	
221.	Trovato 2015	The Resistible Rise of B-Line Lung Ultrasound Artefacts...Reissig A, Copetti R: Lung ultrasound in community-acquired pneumonia and in interstitial lung diseases. Respiration 2014; 87: 179–189.	Review
222.	Trovato 2014	Thoracic ultrasound: Possible complementary criteria for the assessment of pulmonary fibrosis...Hasan AA, Makhlouf HA. B-lines: Transthoracic chest ultrasound signs useful in assessment of interstitial lung diseases. Ann Thorac Med 2014;9:99-103	Letter to the editor
223.	Hasan 2014	Reply to the editor...Volpicelli G, Elbarbary M, Blaivas M, Lichtenstein DA, Mathis G, Kirkpatrick AW, et al. International Liaison Committee on Lung Ultrasound (ILC-LUS) for International Consensus Conference on Lung Ultrasound (ICC-LUS). International e	Letter to the editor

224.	Barskova	S.9.1 Lung ultrasound for the screening of interstitial lung disease in SSc.	Conferance abstract
225.	Jankowich 2008	Heterogeneity in combined pulmonary fibrosis and emphysema.	Case report
226.	Volpicelli 2007	Significance of comet tail artifacts at lung ultrasound...Am J Emerg Med. 2007 Jul;25(6):724-5	Letter to the editor
227.	Chan 2007	Comet tail artifacts in emergency chest ultrasound...Am J Emerg Med. 2006 Oct;24(6):689-96	Letter to the editor
228.	Smargiassi 2016	Possible use of chest ultrasonography in asbestos related lung diseases	Conferance abstract
229.	Quarato 2023	Thoracic ultrasound combined with low-dose computed tomography may represent useful screening strategy in highly exposed population in the industrial city of Taranto (Italy)	Wrong patient population
230.	Manandhar 2023	Lung Ultrasound in Diagnosis of Interstitial Lung Disease	Wrong patient population
231.	MohammadRezaBeigi 2023	Lung ultrasound compared to computed tomography detection and automated quantification of systemic sclerosis-associated interstitial lung disease: preliminary study	Wrong patient population
232.	Joerg 2023	Chest ultrasound findings in usual interstitial pneumonia patterns:a pilot study	Duplicate
233.	Tanten 2023	VALUE OF LUNG ULTRASOUND FOR THE DETECTION OF INTERSTITIAL LUNG DISEASE IN PATIENTS WITH RHEUMATOID ARTHRITIS.	Conferance abstract
234.	Beigi 2023	LUNG ULTRASOUND CHANGES COMPARED WITH AUTOMATED QUANTITATIVE COMPUTED TOMOGRAPHY FOR DETECTING SYSTEMIC SCLEROSIS-ASSOCIATED INTERSTITIAL LUNG DISEASE.	Conferance abstract
235.	Carranza-Enriquez 2023	DIAGNOSTIC ACCURACY OF LUNG ULTRASOUND FOR DETECTING INTERSTITIAL LUNG DISEASE AMONG PATIENTS WITH SYSTEMIC SCLEROSIS: A COMPARATIVE STUDY OF TWO SCANNING PROTOCOLS.	Conferance abstract

236.	DiBattista 2023	LUNG ULTRASOUND FOR THE FOLLOW-UP OF SYSTEMIC SCLEROSIS-RELATED INTERSTITIAL LUNG DISEASE TREATED WITH NINTEDANIB: OUTCOMES AND COMPARISONS WITH OTHER ASSESSMENT TOOLS.	Conferance abstract
237.	Walterspacher 2013	Respiratory muscle function in interstitial lung disease	Unable to contact author
238.	Falcetta 2018	The role of lung ultrasound in the diagnosis of interstitial lung disease	Review
239.	Sperandeo 2019	Transthoracic ultrasound versus intraoperative ultrasound in patients with pulmonary fibrosis: Reappraisal of artifacts	Wrong study design
240.	Decuzzi 2006	Transthoracic ultrasound in the evaluation of pulmonary fibrosis. Our experience	Only Abstract
241.	Vassalou 2017	Modified Lung Ultrasonographic Technique for Evaluation of Idiopathic Pulmonary Fibrosis: Lateral Decubitus	Study population is included in a nother selected
		Position: Lateral	study
242.	Banerjee 2024	Correlation of diaphragmatic mobility and thickening assessed by lung ultrasound with severity of interstitial lung disease	Unable to contact author
243.	MohammadRezaBeigi 2024	Lung ultrasound compared to computed tomography detection and automated quantification of systemic sclerosis-Associated interstitial lung disease: Preliminary study	Wrong patient population
244.	Smargiassi 2020	Possible role of chest ultrasonography for the evaluation of peripheral fibrotic pulmonary changes in patients affected by idiopathic pulmonary fibrosis-pilot case series	Unable to contact author
245.	Zhonghua Jiehe Huxi Zazhi 2022	Research progress in bedside ultrasound assessment of diaphragm function in respiratory diseases	Article is not available
246.	Abdullayeva 2024	The role of lung ultrasound and ultrasound elastography in diagnosis of interstitial lung diseases	Unable to contact author

247.	Symvoulakis 2023	Assessing feasibility of targeted primary care referrals for patients with clinical suspicion of interstitial lung disease using lung ultrasound: A prospective case finding study. The potential benefits of LUS utilization	Wrong patient population
248.	Watanabe 2025	Lung ultrasound in the assessment of interstitial lung disease in patients with connective tissue disease: Performance in comparison with high-resolution computed tomography	Wrong patient population
249.	Kildegaard 2024	Thoracic ultrasound shear wave elastography in interstitial lung disease: an explorative pilot study	Conferance abstract
250.	Otaola 2024	Lung Ultrasound as a Screening Tool for Subclinical Rheumatoid Arthritis-Associated Interstitial Lung Disease	Conferance abstract
251.	Vermant 2024	Lung Ultrasound as a Screening Tool for Subclinical Rheumatoid Arthritis-Associated Interstitial Lung Disease	Conferance abstract
252.	Sofiudottir 2024	Thoracic ultrasound findings and pulmonary diagnosis in rheumatoid arthritis with respiratory symptoms	Conferance abstract
253.	Sahin 2024	Correlation of functional data with diaphragm measurements evaluated by ultrasound in patients with Interstitial Lung Disease	Conferance abstract
254.	Milad 2025	Lung ultrasound for assessing disease progression in UIP and NSIP: a comparative study with HRCT and PFT/DLCO	Wrong study design
255.	Sadaka 2023	Lung ultrasonographic findings and oxygen desaturation as markers of severity in diffuse parenchymal lung diseases	Conferance abstract
256.	Posavec 2023	Pleural irregularity and diaphragm mobility among patients with systemic sclerosis	Conferance abstract
257.	Maria 2024	Performance of Lung Ultrasound as a Screening Tool for Subclinical Rheumatoid Arthritis-Associated Interstitial Lung Disease: a Multicenter Study	Wrong patient population

258.	Ferro 2025	Pleural Irregularities: A new ultrasound marker for lung involvement in primary Sjogren's disease	Wrong study design
259.	DiBattista 2024	Lung ultrasound and high-resolution computed tomography quantitative variations during nintedanib treatment for systemic sclerosis-associated interstitial lung disease	Wrong patient population
260.	Ancuta 2024	B-Lines in the Assessment of Interstitial Lung Disease Associated with Scleroderma: The Role of Handheld Devices	Wrong patient population
261.	Mento 2024	Lung Ultrasound Spectroscopy Applied to the Differential Diagnosis of Pulmonary Diseases: An In Vivo Multicenter Clinical Study	Wrong study design
262.	Cao 2022	Lung Ultrasound Score in the Assessment of Interstitial Lung Disease in Connective Tissue Diseases: Correlation with High-Resolution Computed Tomography	Only Abstract
263.	Zhang 2024	Risk prediction modelling in idiopathic inflammatory myositis-associated interstitial lung disease based on seven factors including serum KL-6 and lung ultrasound B-lines	Wrong study design
264.	Farrag 2023	The Reliability of Ultrasound in Diagnosis of Idiopathic Pulmonary Fibrosis	Conferance abstract
265.	Vermant 2024	EXPLORING POTENTIAL SCREENING TOOLS FOR INTERSTITIAL LUNG DISEASE IN RHEUMATOID ARTHRITIS	Only Abstract
266.	Perandones 2024	VALUE OF LUNG ULTRASONOGRAPHY TO DETECT INTERSTITIAL LUNG DISEASE IN PATIENTS WITH RHEUMATOID ARTHRITIS	Conferance abstract
267.	Sofiudottir 2024	DETECTION OF INTERSTITIAL LUNG DISEASE IN RHEUMATOID ARTHRITIS BY THORACIC ULTRASOUND	Wrong patient population

268.	Tang 2024	RHEUMATOID ARTHRITIS-ASSOCIATED ILD: SCREENING AND EVALUATION IN HIGH-RISK PATIENTS (RAISE) STUDY: PILOT DATA FOR THE POTENTIAL ROLE OF LUNG ULTRASOUND AS A SCREENING TOOL	Conferance abstract
269.	Dashti 2024	Lung quantitative ultrasound to stage and monitor interstitial lung diseases	Wrong patient population
270.	Han 2024	Screening value of lung ultrasound and pleural shear wave elastography in connective tissue disease-related interstitial lung disease: a preliminary study	Wrong study design
271.	Otaola 2024	Lung ultrasound is a promising screening tool to rule out interstitial lung disease in patients with rheumatoid arthritis	Wrong patient population
272.	Santos-Moreno 2024	Usefulness of Lung Ultrasound as a Method for Early Diagnosis of Interstitial Lung Disease in Patients with Rheumatoid Arthritis	Wrong patient population
273.	Mercier 2024	High Prevalence of the Lung Ultrasound Interstitial Syndrome in Systemic Sclerosis Patients with Normal HRCT and Lung Function-A Pilot Study	Wrong patient population
274.	Gutierrez 2024	The Value of Ultrasound for Detecting and Following Subclinical Interstitial	Wrong patient population
		Lung Disease in Systemic Sclerosis	
275.	Vermant 2024	Lung Ultrasound as a Screening Tool in Rheumatoid Arthritis	Wrong patient population
276.	Vermant 2023	Ultrasonographic Presentation and Anatomic Distribution of Lung Involvement in Patients with Rheumatoid Arthritis	Wrong patient population
277.	Nagendra 2023	A COMPARATIVE ANALYSIS OF CHEST ULTRASONOGRAPHY AND HIGH-RESOLUTION CT IN THE DIAGNOSIS AND EVALUATION OF INTERSTITIAL LUNG DISEASES	Wrong patient population
278.	Thapa 2023	Utility of Using Ultrasound and Spirometry in Predicting Transdiaphragmatic Pressure (Pdi) in Patients With Moderate to Severe IIP	Conferance abstract

279.	SantanaPeralta 2023	CORRELATION BETWEEN LUNG ULTRASOUND AND HIGH-RESOLUTION CHEST CT IN SYSTEMIC SCLEROSIS	Conferance abstract
280.	Santana 2016	Diaphragmatic dysfunction evaluated by ultrasound correlates with dyspnea, exercise and quality of life in interstitial lung disease	Conferance abstract
281.	Doveri 2010	Ultrasound lung comets as a long-term prognostic determinant in systemic sclerosis	Only Abstract
282.	Ferro 2024	Pleural Irregularities: A new ultrasound marker for lung involvement in primary Sjogren's disease.	Wrong patient population
283.	Watanabe 2024	Lung ultrasound in the assessment of interstitial lung disease in patients with connective tissue disease: Performance in comparison with high-resolution computed tomography.	Wrong patient population
284.	Wu 2024	[Radiological assessment of interstitial lung disease: what can lung ultrasound do?].	Not able to translate
285.	BaydarToprak 2022	Lung ultrasound in the follow-up of stable idiopathic pulmonary fibrosis	Examined US modalities other than DUS
286.	Lacedonia 2021	The role of transthoracic ultrasound in the study of interstitial lung diseases: High-resolution computed tomography versus ultrasound patterns: Our preliminary experience	Examined US modalities other than DUS
287.	Manolescu 2020	Ultrasound mapping of lung changes in idiopathic pulmonary fibrosis	Examined US modalities other than DUS
288.	Mansour 2018	Sonographic features of idiopathic pulmonary fibrosis	Examined US modalities other than DUS
289.	Sperandeo 2009	Transthoracic Ultrasound in the Evaluation of Pulmonary Fibrosis: Our Experience	Examined US modalities other than DUS
290.	Vassalou 2018	Lung Ultrasonography in Patients With Idiopathic Pulmonary Fibrosis: Evaluation of a Simplified Protocol With High-Resolution Computed Tomographic Correlation	Examined US modalities other than DUS

Table S4: Overview of final judgement of each study based on QUADAS-2 sub-questions.

Study	Patient selection		Index test(s)		Reference Standard		Flow and Timing
	Could the selection of patients have introduced bias?	Concerns that the included patients do not match the review question?	Could the conduct or interpretation of the index test have introduced bias?	Concerns that the index test, its conduct, or interpretation differ from the review question	Could the reference standard, its conduct, or its interpretation have introduced bias?	Concerns that target condition defined by the reference standard does not match review question?	Could the patient flow have introduced bias?
Bernardinello 2023	High	Low	Unclear	Low	Unclear	Unclear	Unclear
Boccatonda 2019	Unclear	Low	Unclear	Low	Unclear	Unclear	Unclear
He 2014	High	Low	High	Low	Low	Low	High
Kismet 2023	High	Low	High	Low	Unclear	Low	Unclear
Milesi 2023	High	Low	High	Low	Unclear	Low	Unclear
Santana 2019	High	Low	High	Low	High	Low	Unclear

Figure S1: Assessment of risk of bias.

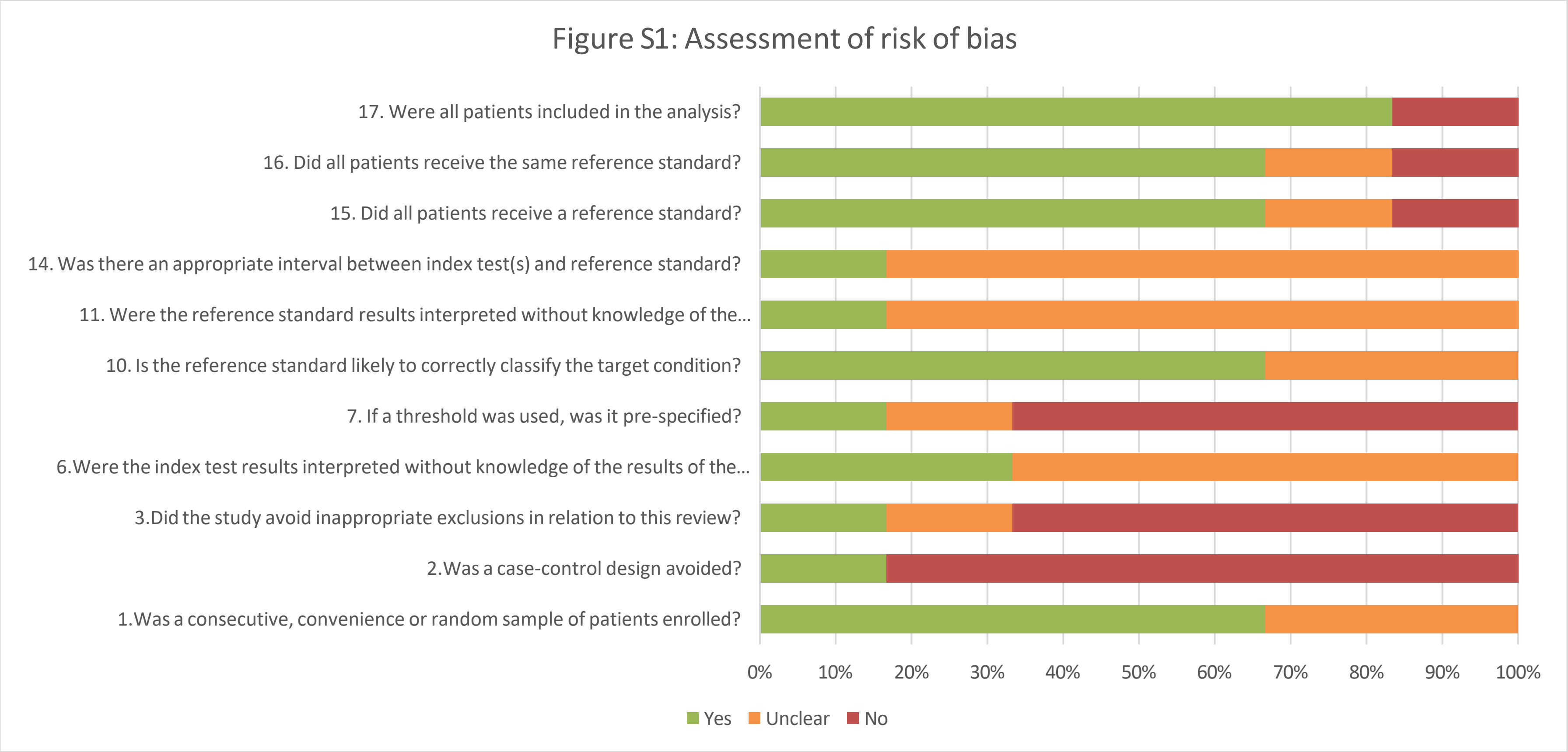

Figure S2: Concerns regarding applicability.

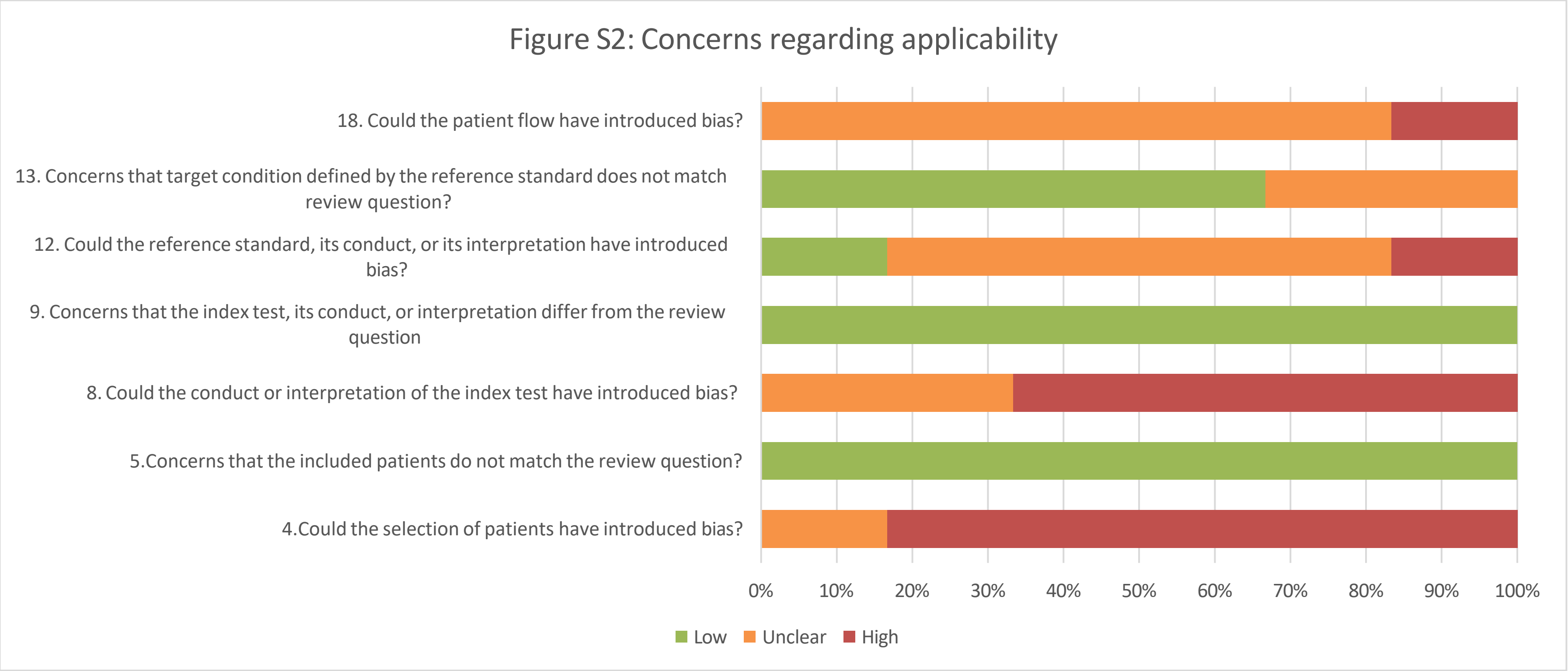

Table S5: Detailed GRADE certainty-of-evidence assessment by outcome

Outcome	Evidence base	Risk of bias	Indirectness	Imprecision	Inconsistency	Publication bias	Overall certainty	Explanation
**Diaphragm displacement / excursion**	6 studies; 166 participants Bernardinello, Boccatonda, He, Kısmet, Milesi and Santana	Serious Downgraded	Not serious No downgrade	Serious Downgraded because of the small total sample	Not serious No downgrade	Unclear; formal assessment was not possible	**Very low (©◯◯◯)**	Observational evidence started at low certainty and was downgraded for risk of bias and imprecision. No upgrading criteria were identified.
**Diaphragm thickness**	4 studies; 136 participants Bernardinello, Kısmet, Milesi and Santana	Serious Downgraded	Not serious No downgrade	Serious Downgraded because of the small total sample	Not serious No downgrade	Unclear; formal assessment was not possible	**Very low (©◯◯◯)**	Observational evidence started at low certainty and was downgraded for risk of bias and imprecision. No upgrading criteria were identified.
**Thickening fraction**	4 studies; 136 participants Bernardinello, Kısmet, Milesi and Santana	Serious Downgraded	Not serious No downgrade	Serious Downgraded because of the small total sample	Not serious No downgrade	Unclear; formal assessment was not possible	**Very low (©◯◯◯)**	Observational evidence started at low certainty and was downgraded for risk of bias and imprecision. No upgrading criteria were identified.

Notes: Certainty was assessed across each DUS outcome. Observational evidence began at low certainty and was downgraded for serious risk of bias and imprecision. Publication bias could not be formally assessed because fewer than 10 studies contributed to each outcome. No upgrading criteria were identified.

Abbreviations: DUS, diaphragmatic ultrasound; GRADE, Grading of Recommendations Assessment, Development and Evaluation.
